# Encoding kirigami bi-materials to morph on target in response to temperature

**DOI:** 10.1038/s41598-019-56118-2

**Published:** 2019-12-20

**Authors:** Lu Liu, Chuan Qiao, Haichao An, Damiano Pasini

**Affiliations:** Department of Mechanical Engineering, McGill University, Macdonald Engineering Building, Room 372, 817 Sherbrooke Street West Montreal, Quebec, H3A 0C3 Canada

**Keywords:** Engineering, Materials science

## Abstract

Shape morphing in response to an environmental stimulus, such as temperature, light, and chemical cues, is currently pursued in synthetic analogs for manifold applications in engineering, architecture, and beyond. Existing strategies mostly resort to active, namely smart or field responsive, materials, which undergo a change of their physical properties when subjected to an external stimulus. Their ability for shape morphing is intrinsic to the atomic/molecular structure as well as the mechanochemical interactions of their constituents. Programming shape changes with active materials require manipulation of their composition through chemical synthesis. Here, we demonstrate that a pair of off-the-shelf passive solids, such as wood and silicone rubber, can be topologically arranged in a kirigami bi-material to shape-morph on target in response to a temperature stimulus. A coherent framework is introduced to enable the optimal orchestration of bi-material units that can engage temperature to collectively deploy into a geometrically rich set of periodic and aperiodic shapes that can shape-match a predefined target. The results highlight reversible morphing by mechanics and geometry, thus contributing to relax the dependence of current strategies on material chemistry and fabrication.

## Introduction

Natural systems often exhibit an effortless propensity to shape morph in response to light, humidity and other environmental stimuli. Conifer cones, for instance, respond to the moisture content of wet or dry environment through the closure or opening of their overlapping scales, thus displaying a capacity for hygroscopic actuation^1^. Heliotropism of sunflowers is another elegant example of response to sunlight, where solar tracking movements enhance the photosynthesis process and increase growth rates^2^. The array of strategies biological systems offer are currently pursued in synthetic analogs through alternative pathways of broad technological diversity^3–9^. Controlled formation of shape morphing has a number of distinct hallmarks, the most notable being spatial reconfigurability delivered post-fabrication, generation of prescribed motions, morphing induced functionalities (such as actuation, amplified extensibility, and folding), and time-dependent control^10–12^. These along with other benefits have so far contributed to brand shape morphing as a topical theme of research with widespread promise of application across the spectrum of technology, such as autonomous robotics^13,14^, smart textiles^15^, shape-shifting metamaterials^12^, minimally invasive devices^16^, drug delivery^17,18^, and tissue engineering^16,19^.

Shape morphing in artificial materials has been demonstrated with a range of external stimuli and field-responsive materials. Swelling, light, temperature, and other cues, are typical triggers in smart solids, i.e. active materials that undergo a change of their physical properties as a result of phase transformations, conformation shifts of their molecular structure and mechanochemical interactions of their constituents. Active materials appear either individually, e.g. shape memory alloys (SMAs)^20^, or in composite formations, e.g. hydrogel composites^4,21^, ferromagnetic materials with localized inclusions of electrically conductive microparticles^22–24^, hybrids with gradation of particle concentrations in given directions^25^, and patterning of anisotropic materials^26–30^ among others. They require a priori synthesis of their composition and molecular architecture. For example, in SMAs shape changes are obtained by programming the transition temperature, and are the net result of an orderly shift (twinning) of a large group of atoms in their crystal lattice, from the austenite to the martensite phase^31^. Shape memory polymers are another example of smart materials. They consist of a polymer network comprising two segregated phases with either covalent cross-link bonds or physical interactions. The switch between them occurs at a temperature programmed through the synthesis process of their polymer network^32^. Hydrogels are also known for their phase-transition properties responsive to a temperature stimulus. Their polymer network consists of covalently cross-linked polymer chains that can aggregate with water to form an elastomeric hydrogel. Here volumetric shrinking, which is exploited for shape shifting, is caused by specific temperature-induced interactions between hydrophilic/hydrophobic segments of the polymer chains and the water molecules^33^. Programming shape shifting with active materials, therefore, involves a tight intertwine between the chemical recipes and the fabrication process used to dispense them. Their typical realizations mainly extend to materials that can be polymerized^5,34^, cross-linked^35–38^, and formulated as customized ink of composites^9,39^. For most of them, morphing is irreversible with some exceptions, such as hydrogel composites, which do exhibit reversibility but slow actuation response^40^. In addition, most active materials, especially shape memory polymers, respond with an on-off switch of deformation at a transition temperature set through chemical recipes, and their performance typically degrades steadily under thermomechanical cycles. This characteristic may pose limits of application in regimes operating with temperature-fluctuating stress, where actuation is sought through successive heating/cooling cycles.

In parallel, complementary routes that use passive solids, either standalone or in combination thereof, exist. Those that resort to a single passive solid have been explored to achieve reconfigurability, deployment^41,42^, folding of planar sheets via origami^43–45^, kirigami^46,47^, and combinations thereof^48^, as well as in 3D tessellations of prismatic unit cells^49,50^. Most of them are periodic with a paucity featuring spatial heterogeneity in flat thin sheets^45^ and textured metamaterials^51^, but they all cannot respond to an external stimulus since an external force is needed to induce morphing.

On the other hand, two or more passive materials have been combined in layouts to attain desired thermal expansion performance^52^. Concepts with distinct coefficient of thermal expansion (CTE) arranged in certain configurations, such as bilayer systems^53,54^, and structural layouts in 2D and 3D, such as compliant^55–57^ as well as stiff topologies^58–62^, can generate responses for given magnitude and directionality of thermal expansion. These realizations, however, consist of individual repeated units with tailored CTE, typically yielding zero or negative values, and cannot generate large global deformation of an ensemble of units that can shape-morph on target. These are characteristics often sought in soft robotics, for example when locomotion is prescribed to trace a specific path, and deployable structures, when the deployed state should match non-classical, e.g. freeform, surfaces.

In this work, we demonstrate large temperature-driven morphing from a pair of passive solids, aperiodically arranged in a kirigami bi-material through a basic fabrication process. Temperature-responsive metaunits and aggregation rules are presented to generate a variety of single-piece metaensembles that can conform to a large number of planar shapes. Soft modes of deformation are individually encoded into the morphology of each unit, and a coherent framework is presented to deterministically predict and optimally program the global shape transformaton of the entire kirigami bi-material. Highlighting the notion of functionality induced by the interplay between geometry and mechanics, this work brings to light reversible shape-shifting from passive solids in response to temperature and contributes to relax the dependence on fabrication parameters and material composition. It also provides freedom to program the characteristics of the shape response, including both abrupt and smooth transitions that can gradually evolve even within a large range of temperature, as opposed to the on-off actuation of active solids that takes place at a given temperature value.

## Methodology

### General framework

**Figure 1:**
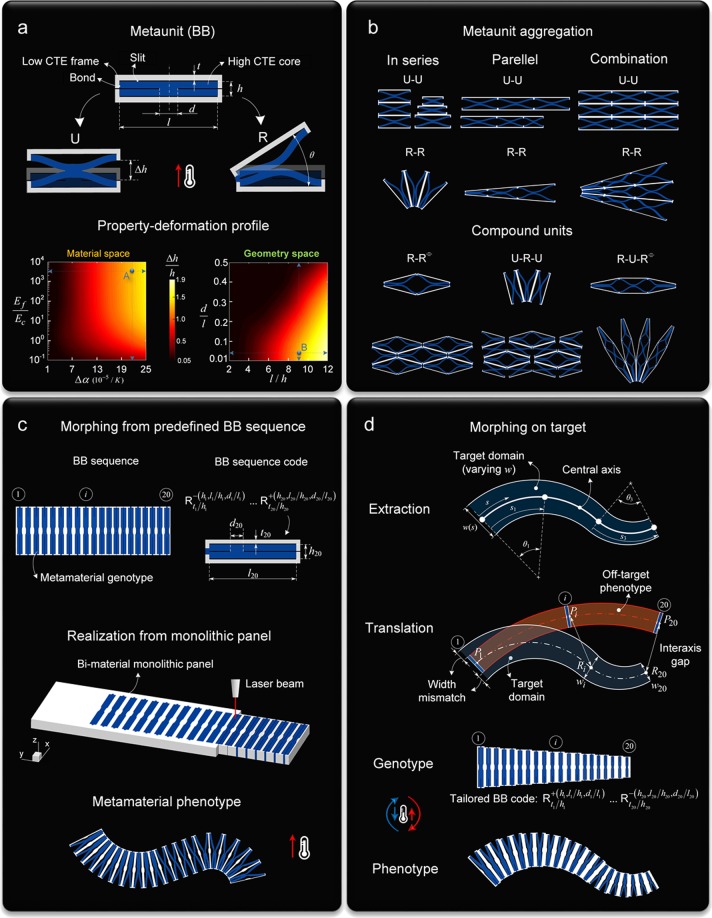
Framework overview for temperature-driven morphing. (**a**) **top**, *Building block (BB) with high and low CTE constituents*. Enforced mid-plane symmetry seals a unidirectional (U) soft mode (Δ*h*/*h*), whereas broken symmetry combined with core-end closure releases a rotational (R) mode (*θ*). (**a**) **bottom**, *Assignment of deformation-property profile*. Attainable range of elastic deformation for BB, measured in the material (Δ*α* = *α*_*c*_ − *α*_*f*_ versus *E*_*f*_/*E*_*c*_ (*f*: frame, *c*: core)) and geometric space (*l*/*h* versus *d*/*l*), at temperature 120 °C for *l*/*h* = 9 and *d*/*l* = 0.05 (point B), and *E*_*f*_/*E*_*c*_ = 3200, *α*_*c*_ − *α*_*f*_ = 210 × 10^−6^/K (point A). (**b**), *Metaunit aggregation*. Pathways for BB periodic and aperiodic aggregates monolithically connected in parallel, series, and combination thereof, from repeated (top) and compound units (bottom) (R^≡^ indicates the reflection of R). (**c**), *Forward problem*. Morphing prediction from predefined sequence of *m* BBs in series (genotype) described through the string ${B}_{t/h}^{\pm (h,\,l/h,d/l)}$, where B stands for U or R, the superscript collects the geometric parameters of the high CTE material, and the subscript those of the low CTE material, +/− indicates direction of rotation for R-BB (+clockwise). Below, laser cut of a single piece bi-material panel and morphed configuration (phenotype). (**d**), *Inverse problem*. Extraction of shape descriptors from target domain, assumed here as arc spline axis, made of G^1^ continuous arcs and straight-line segments, and symmetric boundaries. Translation of shape descriptors into a tailored BB sequence code that enables the central axis and varying width of an off-target phenotype (red) to conform to those of the target domain (blue). Qualitative sketches out of scale at given temperature.

At the roots of our scheme (Fig. 1), there are three basic notions with two routes that enact shape-matching on demand and in a reversible fashion: (i) the definition of a *functional metaunit*, a building block (BB) comprising two passive solids, capable of expressing distinct modes of deformation upon a change in temperature (Fig. 1a, top); (ii) the assignment of a *deformation-property profile* to the BB, which systematically correlates the achievable amplitude of deformation a BB can deliver to its material and geometric attributes (Fig. 1a, bottom); (iii) the provision of *aggregation rules* to adjacent BBs, which enable monolithic tessellations of broad geometric diversity (Fig. 1b). With these notions, access to morphing is through two ports of entry. The first promotes and predicts morphing from a predefined metamaterial architecture (Fig. 1c). The second generates a morphed state that can match a prescribed target (Fig. 1d).

We first start with a descriptive outlook of the salient points underpinning our framework, demonstrating hereafter the details. The metaunit (Fig. 1a) consists of a rigid frame with low coefficient of thermal expansion (CTE) (grey) that encloses a soft core with high CTE (blue), each responding to temperature at a different rate. The former confines the propensity of the latter to volumetrically expand under temperature due to their CTE mismatch. At their vertical edges, the two are fully bonded, whereas a slit appears along the entire length of their horizontal interfaces. The core is partially riven along its horizontal axis of symmetry with a ligament, *d*, connecting the upper and lower parts. By harnessing the position of the core ligament, we can seal onto BB two distinct *deformation modes*. Enforced reflection symmetry across the vertical midplane (dash-line) imprints a unidirectional floppy mode (U), where U-BB resembles an accordion that axially expands by Δ*h*. A loss of symmetry, on the other hand, combined with end core closure instills a rotational (R) mode, where R-BB responds as a clothespin that can open by an angle *θ*.

While the mode of deformation is mainly conferred by topology (U versus R), temperature, as well as materials and geometry of each unit, govern the magnitude of the response. To capture this dependence, we gauge the attainable range of elastic deformation the metaunit can attain at a given temperature upon manipulation of its material and geometric attributes. This defines the *property-deformation profile*, which we cast here in two sets. The first maps the role of materials, Δ*α* = *α*_*c*_ − *α*_*f*_ versus *E*_*f*_/*E*_*c*_ (*f*: frame, *c*: core), and the second that of geometry, *l*/*h* versus *d*/*l*, the groups of parameters that most influence BB response. As an illustrative example, Fig. 1a (bottom) shows both the material and geometry spaces at *T* = 120 °C obtained for a representative U-BB with given materials (Δ*α* = 210 × 10^−6^/K; *E*_*f*_/*E*_*c*_ = 3200, point A coordinates) and geometry (*l*/*h* = 9*; d*/*l* = 0.05, point B coordinates). The former correlates the amount of uniaxial deformation to a change in material properties, while the latter that to a change in its inner architecture. While specific to this example, the *property-deformation profile* provides a systematic route to assess the deformation a BB can render at a given temperature through control of its material and geometric attributes, hence being the key to predict and program deformation at the rank of the metaunit.

At the next level, there are BB aggregates (Fig. 1b), which we aim here to generate from a single piece, a monolithic dual material panel, as opposed to an assembly of individual parts connected together, as described in the following section. The intrinsic characteristics of BB are conducive to the generation of an array of BB aggregates with rich geometric diversity (See Supplementary Movie 1 for illustrative demonstrations). Figure 1b shows a collection of options, among others. Here BBs are shown to form spatially invariant periodic and aperiodic tessellations not only from primitive units, e.g. R-R or U-U (top), but also from hybrid cells, e.g. U-R-U (bottom), that provide access to a diverse set of morphologies. Interaction between adjacent BBs takes place through monolithic connections that impose the way BBs act collectively, e.g. parallel, series and combination thereof, via either the low CTE material (grey), or at a collection of high CTE locations (blue).

With the notions above, we now tackle the morphing problem of an ensemble of BBs along two pathways facing the questions: how to predict, and how to program global transformations. The forward route is depicted in Fig. 1c with a basic example. Shown here is a sequence of 20 BBs of a given pair of materials monolithically connected in series; the goal is to predict their deployment upon a cycle change of temperature. The undeformed state, the metamaterial *genotype*, realized through a purposely conceived simple process, as explained in the following section, is defined by a string of information, the *BB sequence code*. This carries the order and functional instructions that enable cooperative, frustration-free, shape changes with closely matched deformation at the BB interfaces; it fully connotes the collective deformed state of the metamaterial, physically expressed by the *phenotype*. The complimentary route is depicted in Fig. 1d with another illustrative example. The goal here is to program the genotype with a BB sequence code that elicits reconfiguration into a phenotype matching a given target. Two main steps are involved: *extraction* and *translation*. The former retrieves the shape descriptors of the target domain, described here with a central axis and two symmetric boundaries of varying width. The latter acts on the target descriptors to decode a tailored BB sequence for a phenotype that shape-transforms into the target. As detailed later, the underlying rationale is to make the morphed layout of an off-target phenotype, which is assigned with an arbitrary sequence of BBs, conform to the target domain; and we do so by minimizing the gaps between their central axes and their unmatched widths. The result is a tailored BB code that enacts morphing on target upon heating and directs a reversal upon cooling. Details on this and other parts underpinning the framework follow.

### Fabrication of kirigami bi-materials

While typical kirigami materials are cut from a single solid, here we present a fabrication process for bi-material kirigami that is purposely conceived to be as simple as possible while making use of off-the-shelf passive solids. The aim is to emphasize the notion of shape-matching in response to other than mechanical input by mechanics and geometry, and thus to relax the dependence from manufacturing technology and material chemistry, which are key to programming the response of active materials.

**Figure 2:**
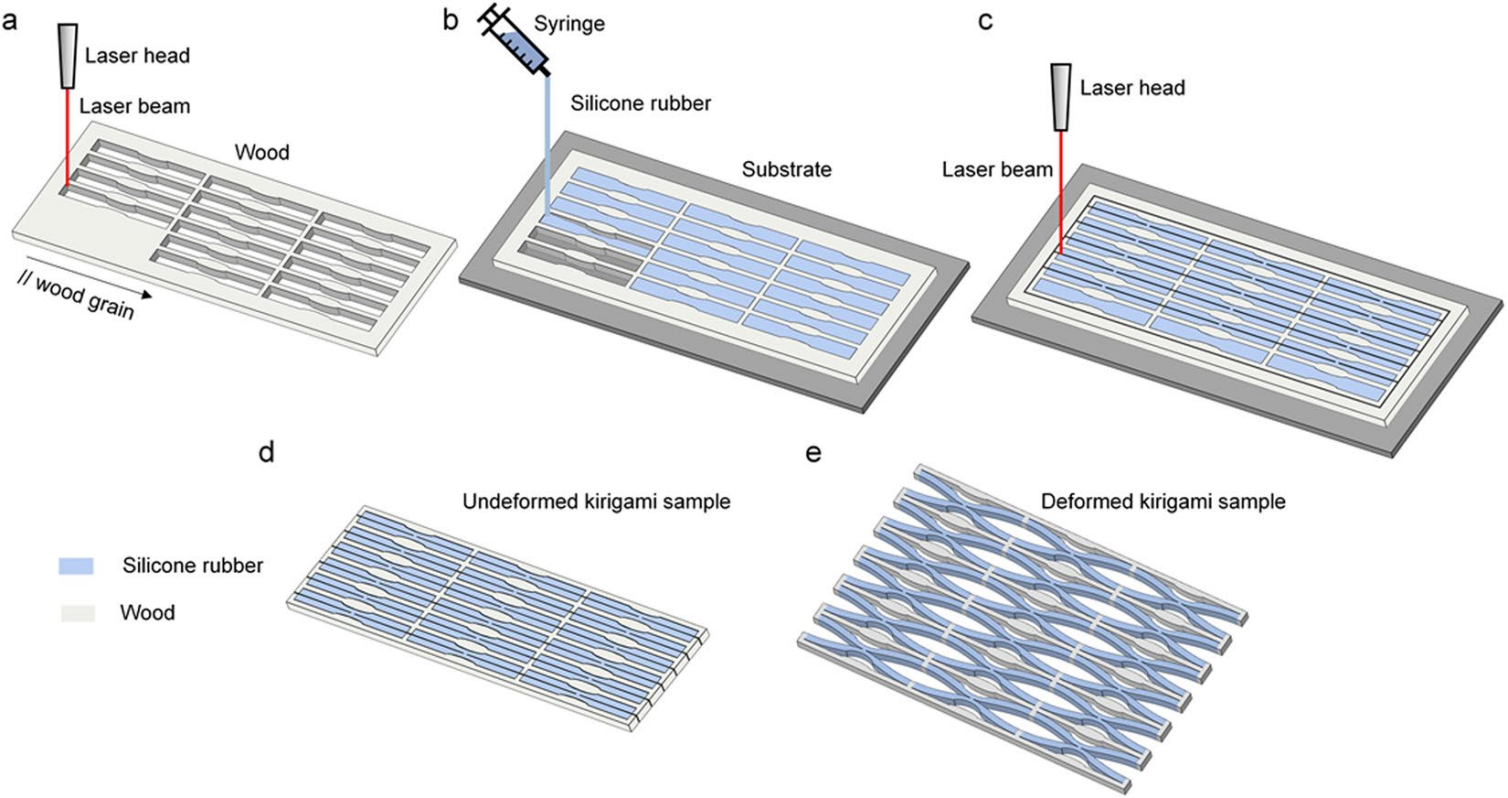
Fabrication process at room temperature. (**a**) Laser cut of a wooden panel forming a mould with an array of voids, each shaped with the geometry of the BB core. (**b**) Casting of silicone rubber filler into the array of voids. (**c**) Laser cut of the kirigami bi-material along the slits of each BB. (**d**) Sample removal from the substrate for the release of a monolithic kirigami bi-material in its undeformed state. (**e**) Deformed kirigami bi-material sample in response to temperature change.

Figure 2 shows the steps describing the realization of an illustrative kirigami specimen comprising 3 by 5 units made of a silicone elastomer (R-2374A silicone rubber compound, Silpak Inc., USA) and hardwood (Black walnut panel, Midwest Products Co., USA), the former representing the high CTE solid and the latter the low CTE. A periodic array of 15 voids aggregated in a hybrid arrangement (3 columns of units in parallel, each with 5 units connected in series), is laser cut (CM1290 laser cutter, SignCut Inc., CA) from a 1/8-inch-thick hardwood panel to create a void-patterned mould subsequently bonded (Instant Adhesive CA4, 3 M Inc., USA) onto a 1/8-inch-thick acrylic substrate. Each void is shaped to host the characteristic geometry of the unit core featuring a semielliptical groove on both its upper and lower edges (Fig. 2a). The silicone elastomer in liquid form is mixed with a platinum-based catalyst to create a cross-linking reaction and then injected to entirely fill the voids of the wooden array. The curing process performed at room temperature for 24 hours turns the silicone elastomer of the building block (BB) core from a liquid into solid (Fig. 2b). During the process, the silicone elastomer bonds to the wooden frame, thus offering the adequate strength for the formation of a monolithic kirigami bi-material. Finally, a laser cutter perforates a set of slits into the kirigami bi-material (Fig. 2c), a step that precedes the sample detachment from the substrate. In the physical specimens, the strait cuts of the BB geometry shown in Fig. 1 are amended with semielliptical slits to facilitate the onset of deformation during experiments. Figure 2d,e show respectively the bi-material kirigami specimen in its undeformed and deformed shape in response to a change of temperature. While this specimen becomes periodically porous with thermal response governed by a single unit, the fabrication process here presented enables the straightforward production of aperiodic kirigami bi-materials with global morphing controlled by the collective response of all the units.

## Results and Discussion

### Metaunit response

**Figure 3:**
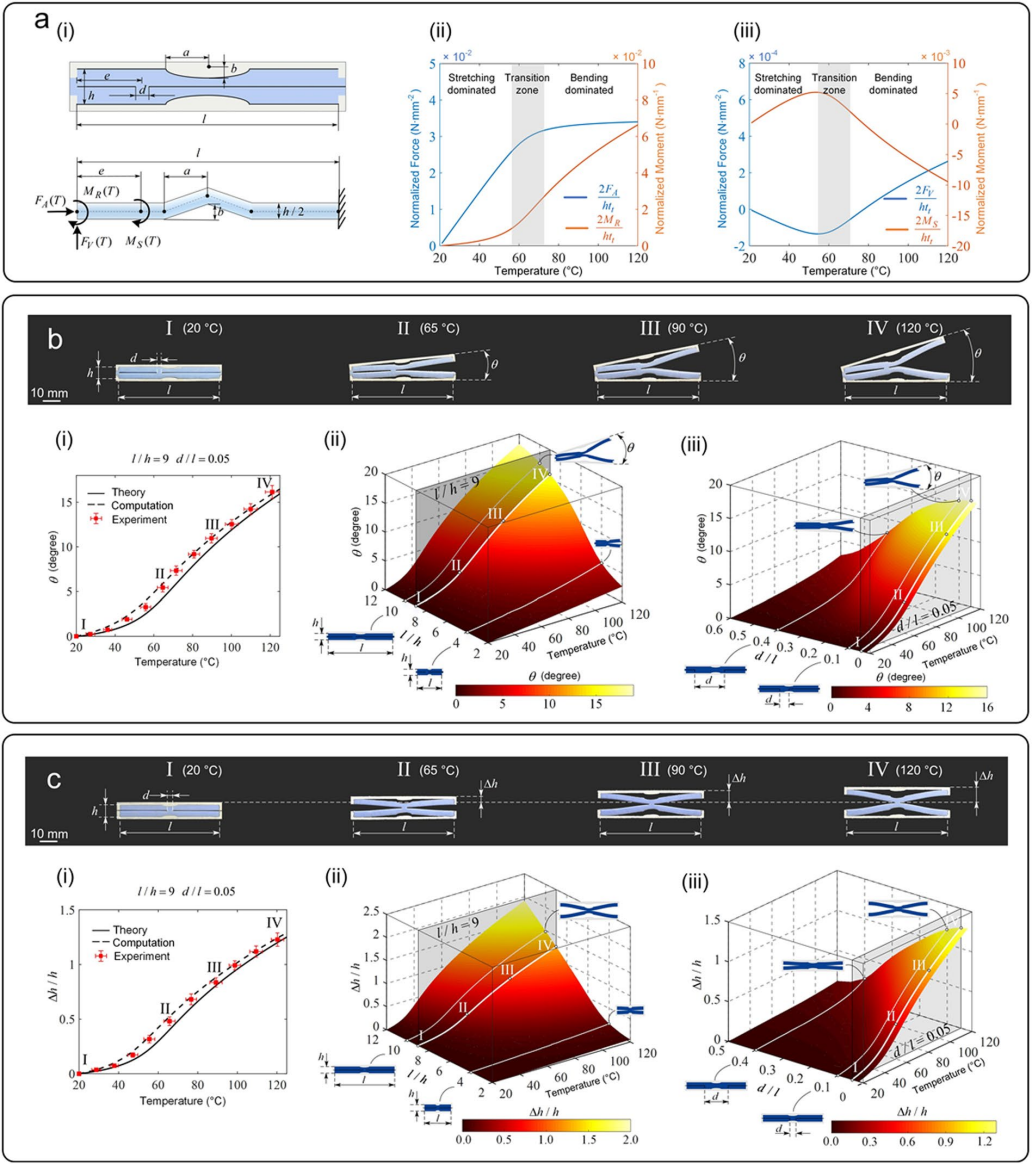
(**a**) Deformation mechanism of BB subject to a uniform thermal field. (i) Geometry of rotational building block in undeformed state with its reduced model, where the high CTE core is condensed to a statically determinate beam-column with reaction and internal force *F*_*A*_, *F*_*V*_ and moment *M*_*S*_ *M*_*S*_. (ii) Evolution of the normalized *FA*, *M*_*R*_, and *F*_*V*_, *M*_*S*_ as a function of temperature solved by Timoshenko’s elastic stability theory. Deformation assessment and evolution of R-BB (**b**) and U-BB (**c**) subject to increasing temperature from 20 to 120 °C. Four deformed states of a representative set of fabricated BBs (*h* = 7 mm, *d/l* = 0.05*, l/h* = 9) with experimental measures of deformation (*θ* and Δ*h/h*) overlaid onto continuous curves obtained through (i) mechanics theory of simplified BB geometry and computation of BB geometry identical to the fabricated samples. (ii) and (iii): prediction maps for *θ* and Δ*h/h* depicting the role of the main geometric descriptors of BB, *l*/*h* and *d*/*l*, as a function of temperature within defined ranges of *l*/*h* and *d*/*l* values. Horizontal and vertical bars indicate the standard deviation around the mean from a pool of measures taken for both temperature and deformation, the former measured at three distinct sites in the heating chamber, the latter obtained from three repeated measurements.

In this section, we use solid mechanics theory to elucidate the deformation response of the kirigami bi-material unit subject to a uniform thermal field for both the unidirectional and rotational floppy modes shown in Fig. 1a. R-BB differs from U-BB for the end closure of the core and a mere symmetry breaking that shifts the position of the connection *d* of the core at an offset *e* from the left end of R-BB. While the underlying mechanism of thermal deformation of both R- and U-BB is caused by the CTE mismatch of the constituents, their topological difference is responsible for each floppy mode. Here we first focus on R-BB in Fig. 3a(i), from which the U-BB response can be derived (see Supplementary S3-a). Due to the symmetry of deformation in R-BB under temperature, we examine the lower half of R-BB, and make the following assumptions: the low CTE frame is rigid with negligible thermal expansion, the length *d* of the connection between the upper and lower portions of the high CTE core is significantly smaller than the BB length ($d\ll l$), and the semielliptical portion of the groove is simplified with two straight inclined beams attached to the horizontal parts (Fig. 3a(i) bottom). Due to the negligible thermal expansion of the low CTE solid, an effective boundary condition is enforced to replace the frame action onto the core which is clamped on both its ends. The reaction from the upper part of R-BB is equivalent to a bending moment (*M*_*S*_) applied at A, i.e. the core connection. The clamped boundary at the left end is released by applying two effective forces (*F*_*A*_, *F*_*V*_) and a moment (*M*_*R*_), all dependent on temperature. The analysis of the building block subjected to uniform temperature is now reduced to the solution of a statically indeterminate problem of a beam-column, which can be solved via Timoshenko’s theory of elastic stability (see Supplementary S3-a). For the elastic properties and CTE (point A in Fig. 1a) of the constituent solids used in the analysis, we used experimentally obtained data (see Supplementary S1 for description of thermal and mechanical testing) with statistical values showing invariance to temperature within the investigated range (see Supplementary S2 for characterization of thermal and mechanical properties).

The results of the theoretical analysis shed light onto the relations of the internal forces (Fig. 3a(ii,iii) and as well as the elastic deflection (Fig. 3b(i)) of R-BB with temperature. The magnitude of *F*_*V*_ and *M*_*S*_ are respectively two and one order lower than *F*_*A*_ and *M*_*R*_ indicating that the transverse force and bending moment at the connection contribute only slightly to the R-BB deformation. Two sequential regimes of deformation can be observed, each controlled by temperature. For low values of temperature, the axial force *F*_*A*_ dominates the bending moment *M*_*R*_ and increases linearly with temperature; here, axial compression governs the R-BB response. With a further increase of temperature, the deformation mode switches through a transition zone above which the axial force flattens at a plateau. Here, BB responds with internal bending with a deformation that is rapid and sensitive to the temperature change at higher values.

The nonlinear response triggered by the core instability can be used to amplify deformation and release it in either a short or long temperature range. Geometric tuning enables temperature to act onto BB not simply as driver of deformation but also as regulator for type (stretch versus bend), magnitude (modest versus large), and gradient of deformation (shallow versus steep). BB can be designed to offer a distinct evolution of deformation that is regulated by temperature with two modes occurring sequentially through a transition zone (around *T* = 60 °C), after which the deformation gradient and the span along which the deformation occurs can be programmed through geometry. In particular, the position of the transition zone can be programmed by manipulating the aspect ratio of the BB core, while the span of the transition zone by the size of the elliptical groove and other geometric parameters (see Supplementary S3-c). This provides a large design space to tune both the deformation gradient and the range of temperature upon which deformation continuously occurs. Deformation can thus be amplified by working near instability, hence generating abrupt changes in response in a short temperature range, or it can be calibrated to ensure a steady and gradual expansion rate over a sizeable temperature span. These characteristics are distinct from those offered by active materials, e.g. shape memory polymers, which through chemistry manipulation can typically shows an on-off switch of deformation at the glass transition temperature.

The experimental response validating the theoretical results are shown in Fig. 3b,c for both R-BB and U-BB. On the top four deformed states (I to IV) are shown each rendered at a given temperature. The corresponding experimental measures of deformation, *θ* and Δ*h*/*h*, are illustrated in the plots below as a function of temperature. Superimposed are also the theoretical results and computational results, both in quantitative agreement. Additional sets of results obtained for units with other dimensions (see Supplementary S3-b) validate the theory with differences between the experimentally measured deformation and theoretical predictions in the high temperature regime below 15%, a value attributed to frictional dissipation accrued during testing as well as the adoption of a simplified structural analog for our theoretical model (see Supplementary S3-a).

Computational models provide further guidance in assessing the role of the geometric attributes that most and least influence BB response. Figure 3a(ii),(iii),b(ii),(iii) show the outcome of harnessing the prime attributes (*h*/*l* and *d*/*l*) of U-BB and R-BB within defined ranges. The maps depict the deformation potential that evolves with temperature, thereby supplementing the maps (Fig. 1a and Supplementary Fig. S9) given for *T* = 120 °C (see Supplementary S3-c for the generation of the deformation-property profile). The longer the BB as well as the smaller the core ligament, the larger the response. Temperature and BB geometry tuning can thus generate a sizeable deformation for a given pair of materials. For example, at *T* = 40 °C, a reduction of *d*/*l* from 0.4 to 0.1 generates a 4.3x gain of Δ*h*/*h* for U-BB, and a 3.2x gain of *θ* for R-BB; these values boost to 4.8x and 1.9x at *T* = 120 °C. Besides *h*/*l* and *d*/*l*, other geometric parameters, such as the size of the BB groove and the offset of the flexural hinge, play a role in the BB response, but the tunability they can offer is quite narrow (see Supplementary S3-c for sensitivity to least influential parameters). Yet as described above, these parameters are effective in calibrating the type and rate of deformation with temperature.

BB integrity and time response are further descriptors of the structural and functional performance of the kirigami unit. The prospect of BB failure involves a balance between the CTE and strain energy of the two constituents, as well as the force of adhesion at their interfaces. Quantitative assessment of the distribution of the interfacial stress (see Supplementary S4 for assessment of bond strength) along with pull-out tests measuring the bonding strength shows a predominant compression state exerted by the BB core onto the enclosing frame with interfaces largely compressed at a magnitude dependent on *h*/*l* and temperature. The analysis shows a sturdy bond at the interfaces with strength value preventing detachment during deformation.

For the temporal response to temperature, the BB deformation is caused by the CTE mismatch of the constituent solids, which do not undergo any atomic or molecular changes, as in the case of active materials. As soon as BB reaches the final temperature, the deformation induced by the internal forces is instantaneous and the mode, magnitude and rate of deformation can be designed through geometry without resorting to chemistry. For shape memory polymers and other active materials, on the other hand, there is a time span involved for the temperature to create configurational changes in the polymers crosslinking. For our kirigami-bimaterials, the time span that we measured to morph does not depend on the properties of the constituent materials, rather mainly on the heating strategy and the experimental setup: the medium surrounding the sample, and the thermal conductivity of the BB surfaces through which the heat transfer takes place. In our experiments, specimens were tested in two heated media, air and oil. Both results showed a deformation evolving over a relatively short period with values (about 5 mins with fan-propelled air and 2 mins in oil bath) in quantitative agreement with those from transient heat transfer analysis (see Supplementary S5).

### Morphing prediction of kirigami bimaterial from given BBs sequence

**Figure 4:**
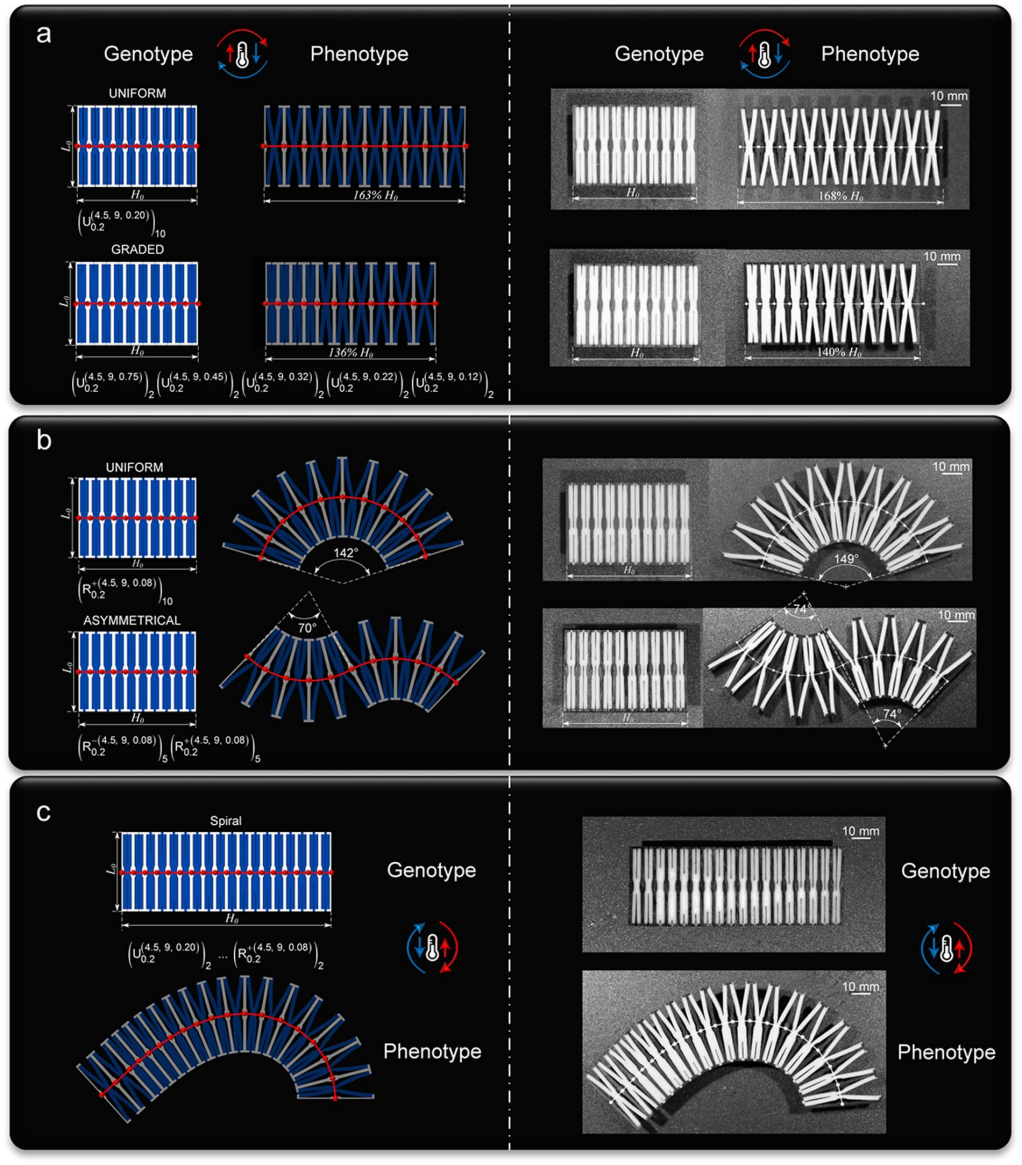
Prediction (left) and experimental (right) validation of a demonstrative array of morphing responses from a series of BBs with predefined sequence, geometry (*h* = 4.5 mm, *l*/*h* = 9, *t*/*h* = 0.2) and materials (wood and silicone rubber). Tested configurations at *T* = 120 °C morphed from their genotype state at room temperature. (**a**) Extensional morphing achieved from a stack of 10 U-BBs: uniform (above) and linear gradation (below) of geometric attributes defined by their BB sequence code. (**b**) Rotational morphing attained from 10 R-BBs in series: uniform distribution of geometric attributes with identical (above) and opposite (below) rotation. (**c**) Spiral morphing predicted and validated from a hybrid sequence of 2 U-BBs and 16 R-BBs. The contents of only two BBs is shown as representatives of the whole BB sequence code. ${B}_{t/h}^{\pm (h,l/h,d/l)}$ indicates BB geometric descriptors (B = U for U-BB, B = R for R-BB). Superscripts for high CTE material ($+$: clockwise rotation for R-BB), subscripts for low CTE material. ${B}_{{t}_{1}/{h}_{1}}^{\pm ({h}_{1},\,{l}_{1}/{h}_{1},{d}_{1}/{l}_{1})}$, $\ldots {B}_{{t}_{i}/{h}_{i}}^{\pm ({h}_{i},{l}_{i}/{h}_{i},{d}_{i}/{l}_{i})}$ connotes a sequence of *i* BBs, and condenses to ${({B}_{t/h}^{\pm (h,l/h,d/l)})}_{m}\,$for a stack of *m* repeating units.

Figure 1c shows a schematic of the forward problem for a monolithic ensemble of BBs stacked in series, each with a predefined set of geometric attributes casted in the BB sequence code. A scheme that uses affine transformations correlates the local deformation of each BB to the global deflection of the phenotype axis and predicts the collective behaviour of BBs at a given temperature (see Supplementary S6 for morphing response from preassigned BB sequence). Its implementation is shown in Fig. 4 on a set of representative genotypes with morphing traits experimentally validated through fabricated samples (*h* = 4.5 mm, *l*/*h* = 9). On the top of Fig. 4a (left) is the simplest case, where a periodic sequence of identical U-BBs is assigned to the genotype, whereas on the bottom there is a stack of BBs with monotonically decreasing *d*/*l*. Similarly, Fig. 4b (left) shows two sequences of R-BB, one sharing prescribed geometric attributes (top), and the other featuring two sets of five R-BBs with opposite direction of rotation (bottom). In Fig. 4c (left), the genotype is dispensed with a BB sequence code defined by a logarithmic spiral. For all three cases, the central (undeflected) axis (red) intersecting the interface mid-points, *P*_*i*_, between adjacent units (red dot) is overlaid on the genotype, and the predicted deflected axis of the phenotype lies on top of the morphed configuration obtained via computations (shaded colour). The counterpart experimental versions are shown on the right of Fig. 4a–c. Here the testing occurred in an oil bath to reduce frictional losses. The relative discrepancy between predictions and experimental measures is below 7% (3% and 2.8% for samples in Fig. 4a, and 5% and 7% for those in Fig. 4b). Overall, the values depicting dimensional differences between genotype and phenotype demonstrate sizable morphing predicted with high-level accuracy. In addition, the experimental results shown in the Supplementary Movies S2–S5 demonstrate fully reversible morphing under the conditions here investigated, i.e. temperature cycle between *T*_room_ and 120 °C.

### Morphing on target via encoded BBs sequence

The response to temperature of our morphable materials can be programmed such that adjacent units can act collectively to reconfigure into a desired form. Here the target to match is a domain (Fig. 1d) with a central axis, an arc spline consisting of G^1^ continuous arcs and straight segments, and two boundaries that are symmetric and continuous with varying width. We match this target by first enforcing equality constraints to guarantee frustration-free motions between adjacent units and inequality constraints that restrict BB deformation within feasible ranges. We then frame these conditions into a constrained optimization problem (see Supplementary S7 for the morphing on target scheme) that mathematically restructures the string of information contained in the BB sequence code of an unprogrammed (off-target) phenotype, which is far from the target because it is randomly assigned with an arbitrary sequence of BBs. Because the central axis and boundaries of the off-target phenotype are incompatible with those of the target domain (Fig. 1d), we minimize the sum of the squares of the distance between their central axes and the mismatched widths of their boundaries.

**Figure 5:**
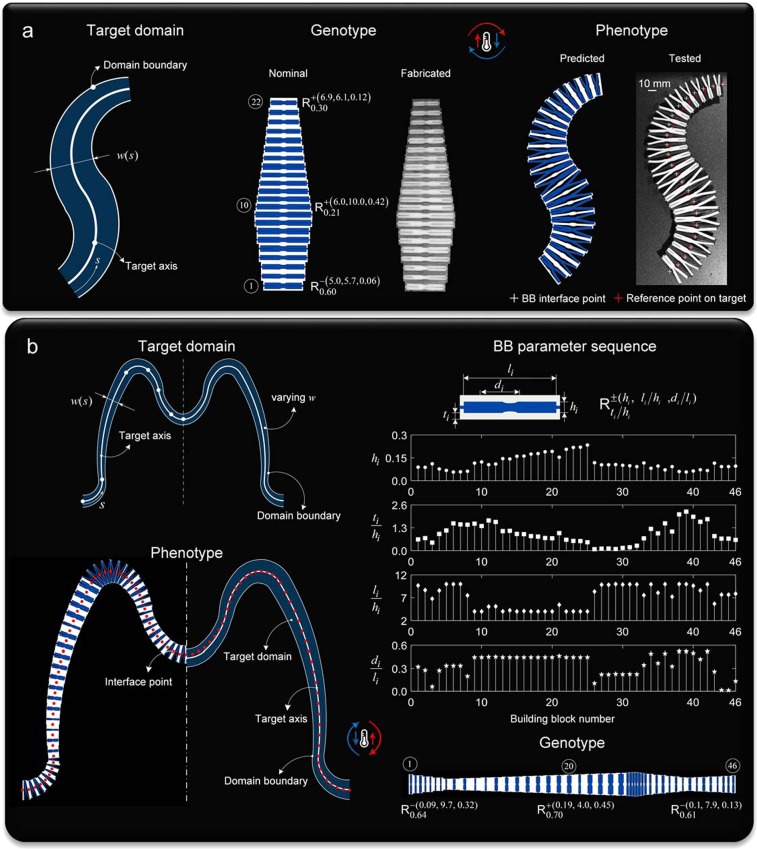
Demonstrations of morphing on target through tailored sequence of metaunits made of hardwood and silicone rubber. (**a**) Target domain of varying width (left) with nominal and fabricated realization of metamaterial genotype comprising 22 BBs (middle) along with predicted and tested configurations of morphed phenotype at *T* = 120 °C (right). (**b**) Domain target of the letter “M” with functions of the axis and varying width to match. 46 units make up the genotype (half is shown) transforming into a phenotype that shape-matches the target with R^2^ = 0.997. The BB parameter sequence shows the stem plot of the optimized values of 4 dimensionless sets of design variables optimized to align the phenotype to the target domain. Below is the genotype (only of shown in the undeformed state) with the BB sequence code given only for representative units, i.e. BB_1_, BB_20_ and BB_46_.

With this scheme, conformal morphing can be realized from several pairs of passive materials. Two illustrative examples (Fig. 5) are demonstrated with two representative materials, wood and silicone rubber. The first (Fig. 5a) shows a simple shape target domain with varying width and an arc spline of two primitives that are G^1^ continuous at their blending points. Here, the BB sequence code of an arbitrary genotype with 22 randomly assigned BBs is decoded to match the shape descriptors of the target. Our morphing scheme restructures the BB sequence code to yield a shape-matching phenotype that is experimentally validated through fabricated samples. A good agreement is observed between the predicted and experimental results. In the second example, the outline of an “M” is chosen as the central axis of the target domain with varying width specified through a set of four continuous functions of varying size along the target axis. With the goal of conforming to the central axis and width of the target, our scheme yields an aperiodic tailored sequence of 46 BBs, which are aperiodic in both their internal and external sizing. This is shown by a stem plot reporting the optimized values of the design variables along with the relevant ratios of the metamaterial genotype at the initial temperature (Fig. 5b and Supplementary S8 and Movie 6). As per the testing results of the “M” shape in Fig. 5b, we note that the limited size of our heating tester prevented us from performing a full-size experiment of the ‘M’ shape sample. Yet, the tested sample in Fig. 5a is representative of the “M” shape because its geometry replicates the varying width domain with a reduced extent, i.e. only 22 building blocks, a requirement that could meet the dimensions of our heating chamber.

## Outlook and Conclusion

**Figure 6:**
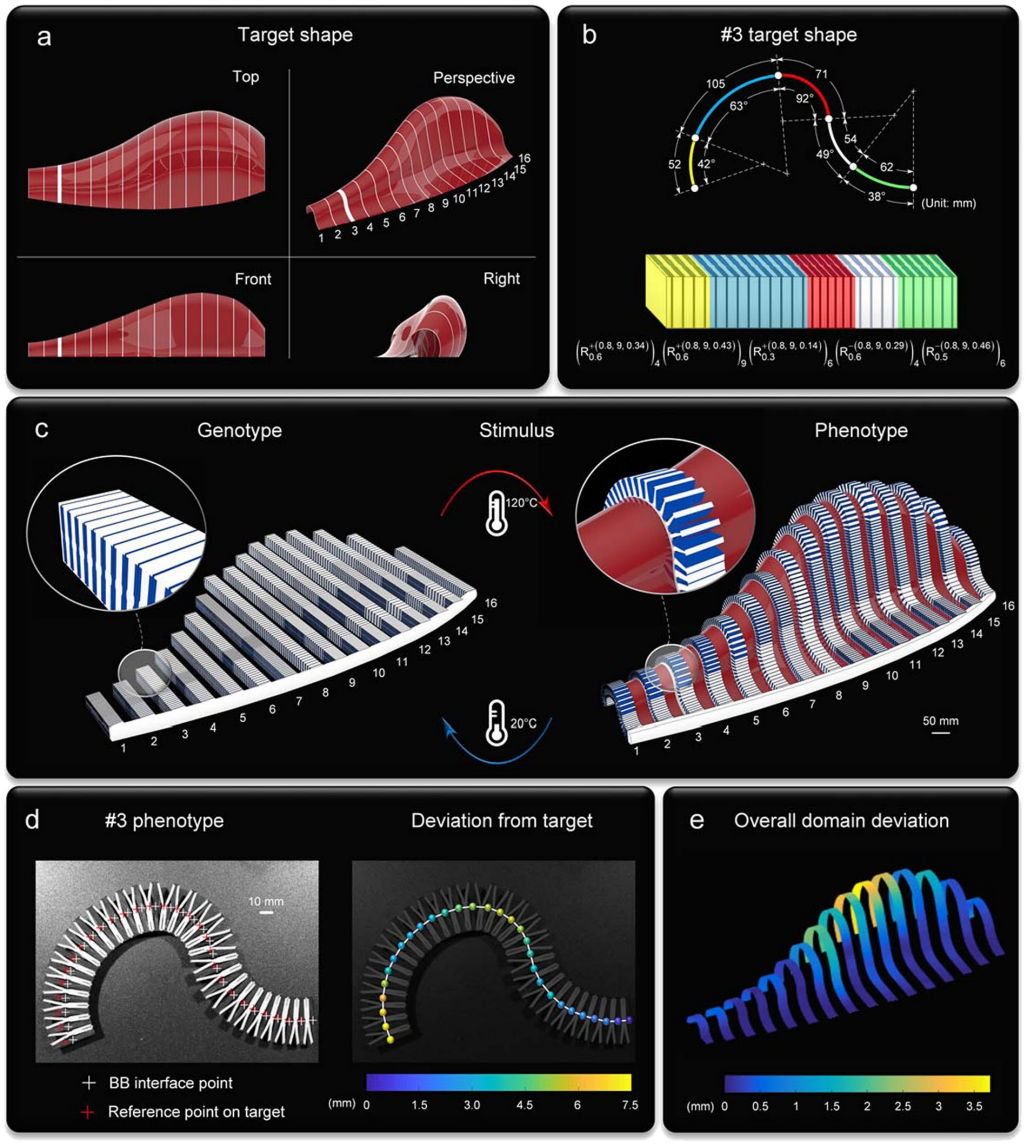
Illustrative example of conformal morphing to a spatial freeform surface. (**a**) Top, front, right and perspective of the target domain: a NURB surface generated from the control points of 16 arc splines used as primitives. (**b**) Extraction (above) of shape descriptors (arc length and opening angle) of #3 primitive, and translation (below) into a conforming genotype defined by its BB sequence code. (**c**) Ensemble of 16 genotypes anchored to a low CTE base (left) and morphed into its phenotype (right); reversible morphing for increasing temperature from 20 °C to 120 °C. (**d**) Morphed configuration (*T* = 120 °C) of #3 phenotype made out of hard wood and silicone rubber: superimposed crosses for points of BB interfaces and target axis (left), and their absolute distance (right). (**e**) Deviation between predicted phenotypes and arc spline targets.

Underpinned by three distinctive notions (Fig. 1), our framework can deterministically predict and precisely impart morphing into a single-piece metamaterial made of passive solids upon a change in temperature. The shape matching of the phenotype to a target domain can be accurately controlled in space through a decoded BB sequence. The constitutive solids are passive, yet their topological arrangement into our metaunit can form aperiodic aggregates that can yield reconfigurations of broad geometric diversity. Figure 6 shows an outlook for our platform applied to drape a three-dimensional surface. Here the target is a spatial freeform surface described by a sweep of 16 arc splines (Fig. 6a), and the goal is to match the shape of the full array of arc splines. Figure 6b shows a representative with its shape descriptors first extracted, and then translated into the BB sequence code. The implementation to all 16 arc splines is depicted in Fig. 6c, where a low CTE cordon monolithically ties them all at the front base, the anterior boundary of the target. Overlaid onto the phenotype ensemble is the target surface (red) with an insert showing precise local conformity (see Supplementary Movie 7). A remarkably good agreement with the target domain and predictions is evinced in Fig. 6d, where experimental results are given for the third arc spline (see Supplementary Movie 8), while Fig. 6e extends the assessment to the global domain.

Despite their shape-shifting promise, there are limits to the approach here presented. First, the deformation of R-BB is an arc whose curvature is dominated by the opening angle and its length scale. If the latter is not constrained, R-BB can in principle capture an arc with any curvature, even one with tiny values resembling a sharp corner. In practice, however, the accuracy and resolution of the manufacturing process might pose restrictions in matching abrupt variations of curvature in the shape target. In addition, the proof-of-concepts tested in this work feature a trade-off between thermal and mechanical performance. In Fig. 6c, gravity was not considered in the morphing direction, since an additional body force could collapse the compliant kirigami material. The low stiffness is typically of kirigami concepts, where the open cuts naturally generate a severe penalty on the material resistance to deformation. Strategies to create stiff and durable kirigami have been proposed and could be implanted here^63^. Another approach is to change the base materials, here hardwood and silicone rubber, as well as the aspect ratio of BB, here quite large (*l*/*h* = 9). For example, a change of silicone rubber, the core material, with a solid of higher elasatic modulus yet distinct CTE, such as polytetrafluoroethylene (PTFE)^64^, and the selection of less slender BB can contribute to compensate the low stiffness observed in our proof-of-concepts. A third option to enhance stiffens in the deformed state is to envision an interlocking strategy that autonomously locks the material phenotype. While these seem promising paths to follow, further work is required to explore them.

The kirigami concepts here presented are complementary yet distinct to the morphing routes currently pursued with active materials. For shape memory polymers and other smart materials, the programming stage of the deformation response goes through a molecular design of their polymer network architectures which are either chemically or physically crosslinked. Through a synthesis process, the switching temperature is programmed, and in a small range of values nearby that temperature, the deformation is fully released with a switch-type response. In addition, the performance of shape memory polymers often worsens under thermomechanical cycles. The kirigami concepts here presented, on the other hand, do no require chemical, rather geometric, strategies applicable to several pairs of off-the-shelf solids including metals. If needed, the selection of the base materials can address the requirement of robustness to fluctuating thermal stress. In addition, the rational manipulation of their geometry, such as the size of the BB groove and the offset of the flexural hinge, allows to calibrate both the rate of deformation and the temperature range within which the response occurs. This geometric tuning offers significant freedom to generate desired types of response, including both sudden and smooth deformation, which could be gradually dispensed even over a large temperature span.

There are a number of potential applications for shape-matching materials across multiple sectors, especially where folding, packaging, and conformational changes are paramount requirements to meet^65^, such as self-reconfigurable medical devices^7,66^, drug delivery systems^18,33^, autonomous soft robotics^67^, and conformable stretchable electronics^68^. The advantages of the concepts here introduced can be capitalized in two primary applications. The first targets repeated and reversible reconfigurability in extreme climates on Earth and in space. Here the transportation of components is typically required in a flat configuration, the deployment is to occur *in-situ*, such as unfolding shelters in unsafe settings^69,70^ or reconfigurable antennas in space^71,72^, and reconfigurability can entail multiple loops of closure and opening, each controlled by temperature cycles. In these conditions, shape memory polymers and other active materials might not be the best fit, not only because their response is typically irreversible, but also because thermomechanical cycles can steadily decrease their performance. The second application is thermal management. Besides shape morphing, our concepts can be programmed to feature adaptive change in their out-of-plane porosity in response to temperature change. The transformation from a fully solid to a fully porous state through temperature change can bring about a large area of voids for heat exchange, conditions that can become an asset for cooling and thermal regulation.

Overall, our framework engages a fine interplay between geometry and mechanics of metaunits to enact morphing in response to temperature. It requires neither manipulation of constituent compositions nor chemical processes. It can predict local and global morphing, as well as reconfigure the morphology of aperiodic architectures into predefined targets. Reversibility through temperature is one of its assets, along with the passive nature of the constituents, and the elimination of external power and control. A large design freedom to tune the thermal response (type, magnitude and rate of deformation) is at hand through manipulation of the internal architecture. Other pairs of passive solids including metals can in principle be used, as long as they offer a sizable distinction in CTE. Purposely implemented with simple yet efficient means of fabrication, our platform is well-suited to other technologies, e.g. multi-material 3D printing^8,73^, offers routes for upscaling and downscaling, and can be also extended to active materials and other stimuli.

## Supplementary information

Supplementary information

Movie 1

Movie 2

Movie 3

Movie 4

Movie 5

Movie 6

Movie 7

Movie 8

## Data Availability

The data that support the findings of this work are available from the corresponding author upon request.

## References

[1] Oliver K, Seddon A, Trask RS (2016). Morphing in nature and beyond: a review of natural and synthetic shape-changing materials and mechanisms. Journal of Materials Science.

[2] Yang H (2017). 3D printed photoresponsive devices based on shape memory composites. Advanced Materials.

[3] Ratna D, Karger-Kocsis J (2008). Recent advances in shape memory polymers and composites: a review. Journal of Materials Science.

[4] Erb RM, Sander JS, Grisch R, Studart AR (2013). Self-shaping composites with programmable bioinspired microstructures. Nature communications.

[5] Harke B (2013). Polymerization inhibition by triplet state absorption for nanoscale lithography. Advanced Materials.

[6] Ge Q, Dunn CK, Qi HJ, Dunn ML (2014). Active origami by 4D printing. Smart Materials and Structures.

[7] Xu S (2015). Assembly of micro/nanomaterials into complex, three-dimensional architectures by compressive buckling. Science.

[8] Ge Q (2016). Multimaterial 4D printing with tailorable shape memory polymers. Scientific reports.

[9] Gladman AS, Matsumoto EA, Nuzzo RG, Mahadevan L, Lewis JA (2016). Biomimetic 4D printing. Nature materials.

[10] Haghpanah B, Salari‐Sharif L, Pourrajab P, Hopkins J, Valdevit L (2016). Multistable Shape‐Reconfigurable Architected Materials. Advanced Materials.

[11] Bertoldi K, Vitelli V, Christensen J, van Hecke M (2017). Flexible mechanical metamaterials. *Nature Reviews*. Materials.

[12] van Manen T, Janbaz S, Zadpoor AA (2018). Programming the shape-shifting of flat soft matter. Materials Today.

[13] Zykov V, Mytilinaios E, Adams B, Lipson H (2005). Robotics: Self-reproducing machines. Nature.

[14] Felton S, Tolley M, Demaine E, Rus D, Wood R (2014). A method for building self-folding machines. Science.

[15] Hu J, Meng H, Li G, Ibekwe SI (2012). A review of stimuli-responsive polymers for smart textile applications. Smart Materials and Structures.

[16] Randall CL, Gultepe E, Gracias DH (2012). Self-folding devices and materials for biomedical applications. Trends in biotechnology.

[17] Tomatsu I, Peng K, Kros A (2011). Photoresponsive hydrogels for biomedical applications. Advanced drug delivery reviews.

[18] Fernandes R, Gracias DH (2012). Self-folding polymeric containers for encapsulation and delivery of drugs. Advanced drug delivery reviews.

[19] Fedorovich NE (2007). Hydrogels as extracellular matrices for skeletal tissue engineering: state-of-the-art and novel application in organ printing. Tissue engineering.

[20] Lagoudas, D. C. *Shape memory alloys: modeling and engineering applications*. (Springer, 2008).

[21] Jeon S-J, Hauser AW, Hayward RC (2017). Shape-morphing materials from stimuli-responsive hydrogel hybrids. Accounts of chemical research.

[22] Mishra SR, Dickey MD, Velev OD, Tracy JB (2016). Selective and directional actuation of elastomer films using chained magnetic nanoparticles. Nanoscale.

[23] Ramachandran V, Bartlett MD, Wissman J, Majidi C (2016). Elastic instabilities of a ferroelastomer beam for soft reconfigurable electronics. Extreme Mechanics Letters.

[24] Kim Y, Yuk H, Zhao R, Chester SA, Zhao X (2018). Printing ferromagnetic domains for untethered fast-transforming soft materials. Nature.

[25] Liu Y (2012). Programmable responsive shaping behavior induced by visible multi-dimensional gradients of magnetic nanoparticles. Soft Matter.

[26] Guan J, He H, Hansford DJ, Lee LJ (2005). Self-folding of three-dimensional hydrogel microstructures. The Journal of Physical Chemistry B.

[27] Stoychev G, Turcaud S, Dunlop JW, Ionov L (2013). Hierarchical multi‐step folding of polymer bilayers. Advanced Functional Materials.

[28] Tolley MT (2014). Self-folding origami: shape memory composites activated by uniform heating. Smart Materials and Structures.

[29] Egunov A, Korvink J, Luchnikov V (2016). Polydimethylsiloxane bilayer films with an embedded spontaneous curvature. Soft matter.

[30] Stoychev G, Guiducci L, Turcaud S, Dunlop JW, Ionov L (2016). Hole‐Programmed Superfast Multistep Folding of Hydrogel Bilayers. Advanced Functional Materials.

[31] Mohd Jani J, Leary M, Subic A, Gibson MA (2014). A review of shape memory alloy research, applications and opportunities. Materials & Design (1980-2015).

[32] Behl, M. & Lendlein, A. Shape‐memory polymers. *Kirk‐Othmer Encyclopedia of Chemical Technology*, 1–16 (2000).

[33] Qiu Y, Park K (2001). Environment-sensitive hydrogels for drug delivery. Advanced drug delivery reviews.

[34] Bauhofer AA (2017). Harnessing Photochemical Shrinkage in Direct Laser Writing for Shape Morphing of Polymer Sheets. Advanced Materials.

[35] Jamal M, Zarafshar AM, Gracias DH (2011). Differentially photo-crosslinked polymers enable self-assembling microfluidics. Nature communications.

[36] Chen C-M, Reed JC, Yang S (2013). Guided wrinkling in swollen, pre-patterned photoresist thin films with a crosslinking gradient. Soft Matter.

[37] Jamal M (2013). Bio‐origami hydrogel scaffolds composed of photocrosslinked PEG bilayers. Advanced healthcare materials.

[38] Na JH (2015). Programming reversibly self‐folding origami with micropatterned photo‐crosslinkable polymer trilayers. Advanced Materials.

[39] Ge Q, Qi HJ, Dunn ML (2013). Active materials by four-dimension printing. Applied Physics Letters.

[40] Ionov L (2014). Polymeric actuators. Langmuir.

[41] Filipov ET, Tachi T, Paulino GH (2015). Origami tubes assembled into stiff, yet reconfigurable structures and metamaterials. Proceedings of the National Academy of Sciences.

[42] Faber JA, Arrieta AF, Studart AR (2018). Bioinspired spring origami. Science.

[43] Hawkes E (2010). Programmable matter by folding. Proceedings of the National Academy of Sciences.

[44] Silverberg JL (2014). Using origami design principles to fold reprogrammable mechanical metamaterials. science.

[45] Dudte LH, Vouga E, Tachi T, Mahadevan L (2016). Programming curvature using origami tessellations. Nature materials.

[46] Blees MK (2015). Graphene kirigami. Nature.

[47] Hwang D-G, Bartlett MD (2018). Tunable Mechanical Metamaterials through Hybrid Kirigami Structures. Scientific Reports.

[48] Castle T (2014). Making the cut: Lattice kirigami rules. Physical review letters.

[49] Overvelde JT (2016). A three-dimensional actuated origami-inspired transformable metamaterial with multiple degrees of freedom. Nature communications.

[50] Overvelde JT, Weaver JC, Hoberman C, Bertoldi K (2017). Rational design of reconfigurable prismatic architected materials. Nature.

[51] Coulais C, Teomy E, de Reus K, Shokef Y, van Hecke M (2016). Combinatorial design of textured mechanical metamaterials. Nature.

[52] Sigmund O, Torquato S (1997). Design of materials with extreme thermal expansion using a three-phase topology optimization method. Journal of the Mechanics and Physics of Solids.

[53] Lakes R (2007). Cellular solids with tunable positive or negative thermal expansion of unbounded magnitude. Applied Physics Letters.

[54] Ha CS, Hestekin E, Li J, Plesha ME, Lakes RS (2015). Controllable thermal expansion of large magnitude in chiral negative Poisson’s ratio lattices. physica status solidi (b).

[55] Jefferson G, Parthasarathy TA, Kerans RJ (2009). Tailorable thermal expansion hybrid structures. International Journal of Solids and Structures.

[56] Wang Q (2016). Lightweight Mechanical Metamaterials with Tunable Negative Thermal Expansion. Physical Review Letters.

[57] Yamamoto N (2014). Thin Films with Ultra-low Thermal Expansion. Advanced Materials.

[58] Xu H, Pasini D (2016). Structurally Efficient Three-dimensional Metamaterials with Controllable Thermal Expansion. Scientific Reports.

[59] Xu H, Farag A, Pasini D (2018). Routes to program thermal expansion in three-dimensional lattice metamaterials built from tetrahedral building blocks. Journal of the Mechanics and Physics of Solids.

[60] Steeves CA (2007). Concepts for structurally robust materials that combine low thermal expansion with high stiffness. Journal of the Mechanics and Physics of Solids.

[61] Wei K, Chen H, Pei Y, Fang D (2016). Planar lattices with tailorable coefficient of thermal expansion and high stiffness based on dual-material triangle unit. Journal of the Mechanics and Physics of Solids.

[62] Lehman J, Lakes RS (2014). Stiff, strong, zero thermal expansion lattices via material hierarchy. Composite Structures.

[63] Shang X, Liu L, Rafsanjani A, Pasini D (2017). Durable bistable auxetics made of rigid solids. Journal of Materials Research.

[64] Xu H, Farag A, Pasini D (2017). Multilevel hierarchy in bi-material lattices with high specific stiffness and unbounded thermal expansion. Acta Materialia.

[65] Zhang Y (2017). Printing, folding and assembly methods for forming 3D mesostructures in advanced materials. Nature Reviews Materials.

[66] Kuribayashi K (2006). Self-deployable origami stent grafts as a biomedical application of Ni-rich TiNi shape memory alloy foil. Materials Science and Engineering: A.

[67] Han Daehoon, Farino Cindy, Yang Chen, Scott Tracy, Browe Daniel, Choi Wonjoon, Freeman Joseph W., Lee Howon (2018). Soft Robotic Manipulation and Locomotion with a 3D Printed Electroactive Hydrogel. ACS Applied Materials & Interfaces.

[68] Rogers J, Huang Y, Schmidt OG, Gracias DH (2016). Origami MEMS and NEMS. Mrs Bulletin.

[69] Verge, A. S. Rapidly deployable structures in collective protection systems. (Army Soldier and Biological Chemical Command Natick MA, 2006).

[70] Mejia-Ariza, J., Murphey, T. & Pollard, E. In *47th AIAA/ASME/ASCE/AHS/ASC Structures, Structural Dynamics*, *and Materials Conference 14th AIAA/ASME/AHS Adaptive Structures Conference 7th* (1686).

[71] Miura K (1985). Method of packaging and deployment of large membranes in space. Title The Institute of Space and Astronautical Science Report.

[72] Moon, F. & Abel, J. Nonlinear dynamics of deployable and maneuverable space structures. (CORNELL UNIV ITHACA NY, 1993).

[73] Sugavaneswaran M, Arumaikkannu G (2014). Modelling for randomly oriented multi material additive manufacturing component and its fabrication. Materials & Design (1980-2015).

