# Supplementary material for: Encoding kirigami bi-materials to morph on target in response to temperature
Source: Sci Rep. 2019 Dec 20;9:19499. doi: 10.1038/s41598-019-56118-2 (PMC6925198; doi:10.1038/s41598-019-56118-2)
Supplement: Supplementary file 1 — Supplementary information [file 41598_2019_56118_MOESM1_ESM.pdf]

SUPPLEMENTARY INFORMATION

Encoding kirigami bi-materials to morph on target in response to temperature

Lu Liu, Chuan Qiao, Haichao An, Damiano Pasini *

Department of Mechanical Engineering, McGill University, Macdonald Engineering Building, Room 372, 817 Sherbrooke Street West
Montreal, Quebec, H3A 0C3, Canada, Tel. 514 398 6295

* e-mail: Damiano.pasini@mcgill.ca

S1. Testing apparatus

Supplementary Fig. S1 shows the schematic of the experimental set-up built to test the thermal deformation of the fabricated metamaterials. The heating chamber consisted of two 200-Watt strip heaters (McMaster-Carr, USA) placed underneath the testing plate, where the temperature was adjusted through a proportion-integration-differentiation (PID) controller (CN7800, OMEGA, USA & Canada). A data acquisition system (cDAQ-9174, National Instruments, USA) was used to collect the temperature values from the thermocouples placed at different locations in the heating chamber with the goal of assessing any instance of temperature heterogeneity throughout the heating chamber. A borosilicate glass cover was placed on the top of the testing system to provide a thermal insulation shield. Specimens were tested both in air and oil, the latter to provide a uniform source of heating and eliminate frictional dissipation between the specimen and the testing plate.

Digital Image Correlation was used to capture the full-field displacement and effective thermal strain of the specimen at increasing levels of temperature. Supplementary Fig. S2 illustrates a representative set of testing on a periodic metamaterial with compound units. Any rigid body movement of the specimen was prevented by anchoring one point (highlighted in red in Supplementary Fig. S2a) of the specimen to the testing plate. Two pairs of black speckles were applied on the rigid frame to trace their movement. Temperature gradually increased from 20 to 120 ℃. Sample deformation was first captured by a digital camera (EOS Rebel T6i, Canon USA), and then processed with a correlation algorithm (Vic-2D, Correlated Solutions Inc., USA) that provided the full-field displacement and strain data between pairs of black speckles in the deformed specimen. The effective thermal strains (ε_{yy}^{*}) was then obtained from the relative displacement normalized by the initial distance between pairs of black speckles. As an illustrative example, Supplementary Fig. S2 on the bottom of each of the six snapshots provides the ε_{yy}^{*} values at two specimen locations for varying levels of temperature.

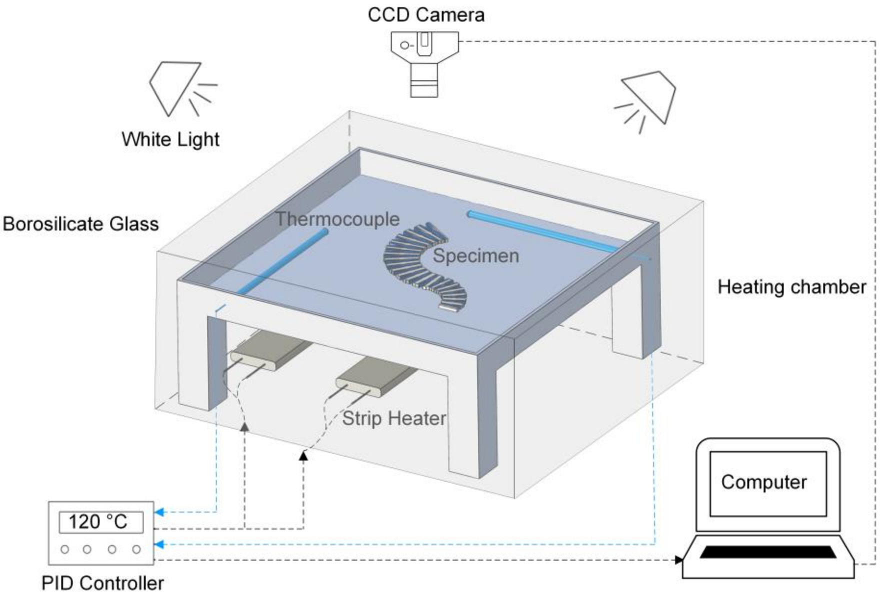

Supplementary Fig. S1: Schematic of the experimental set-up.

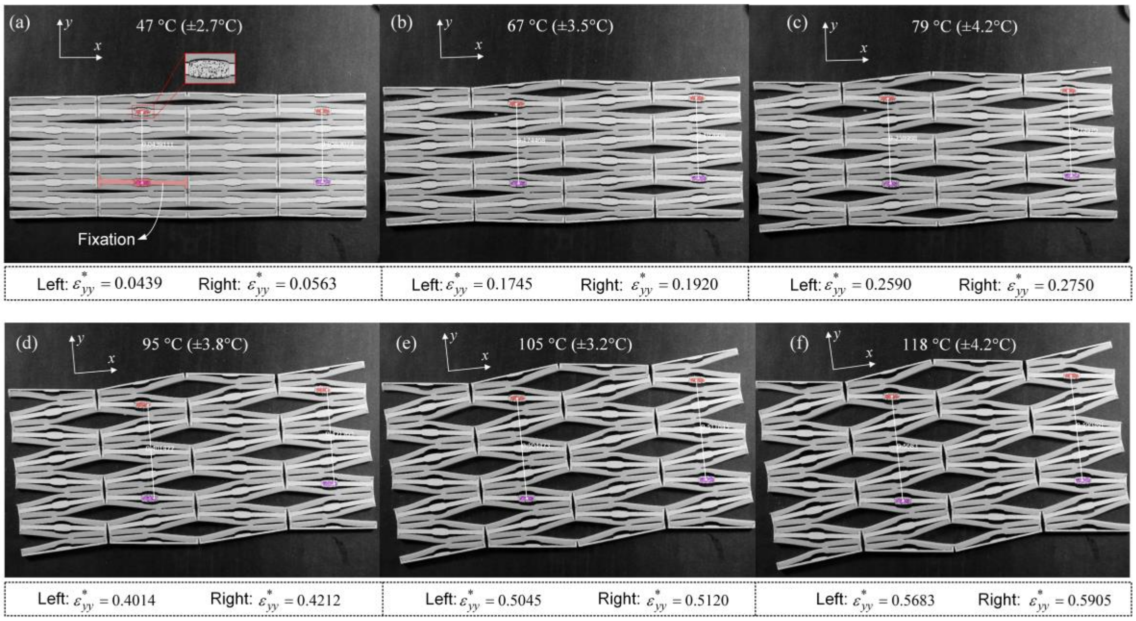

Supplementary Fig. S2: Illustrative snapshots of thermal deformation measures at given values of temperature. Effective values of thermal strain ε^*_{yy} obtained from measurements between pairs of elliptical regions with speckles dispensed on the wooden frame (see insert on the top of (a)).

S2. Characterization of constituent properties: thermal expansion coefficient and Young's modulus

A thermomechanical analyzer (TMA Q400, TA Instruments Inc., USA) was used to measure the coefficient of thermal expansion (CTE) of the constituent solids, silicone rubber and hardwood. For the latter, the CTE was measured in both directions, parallel and perpendicular to the wood grain. Supplementary Fig. S3a reports the CTE for both materials with measured values ($\alpha_{rubber} = 215 \times 10^{-6} K^{-1}$, $\alpha_{//grain} = 5 \times 10^{-6} K^{-1}$, and $\alpha_{\perp grain} = 32 \times 10^{-6} K^{-1}$) almost constant throughout the temperature range under investigation (20 to 120 ℃).

To assess the tensile elastic modulus of the constituents, we tested a set of laser-cut dumbbell-shaped specimens (Supplementary Fig. S3b) with an Instron tensile tester (5982 Series Universal Testing Systems, Instron Inc., USA). For silicone rubber, 3 uniaxial tensile tests were performed under displacement control, from which the most representative nominal stress-strain curve was obtained. To capture the experimental measures, a first order Ogden model was adopted (Supplementary Fig. S3c) with strain energy function given by:

$$W(\lambda_1, \lambda_2, \lambda_3) = \frac{\mu}{\alpha}(\lambda_1^\alpha + \lambda_2^\alpha + \lambda_3^\alpha - 3) \tag{1}$$

where μ and α are the material constants chosen to fit the experimental data (in this case $\mu = 1.03$ and $\alpha = 6.20$), and $\lambda_1, \lambda_2,$ and λ_3 denotes the principal stretches. In addition, the elastic modulus of silicone rubber was extracted from the curve at 25% strain ($E_{25\%} = 3.7$ MPa) and used in our analyses.

For the elastic response of wood, uniaxial tensile tests were performed on specimens with orientation either parallel or perpendicular to the wood grain direction (Supplementary Fig. S3b). Ten dumbbell-shaped specimens were laser cut from a 1/8-inch-thick sheet of hardwood. Supplementary Fig. S3d reports the representative stress-strain curves of the experiments, from which we extracted the following mean values for the Young's moduli: $E_{//grain} = 12.0 \pm 0.3$ GPa, and $E_{\perp grain} = 0.97 \pm 0.09$ GPa.

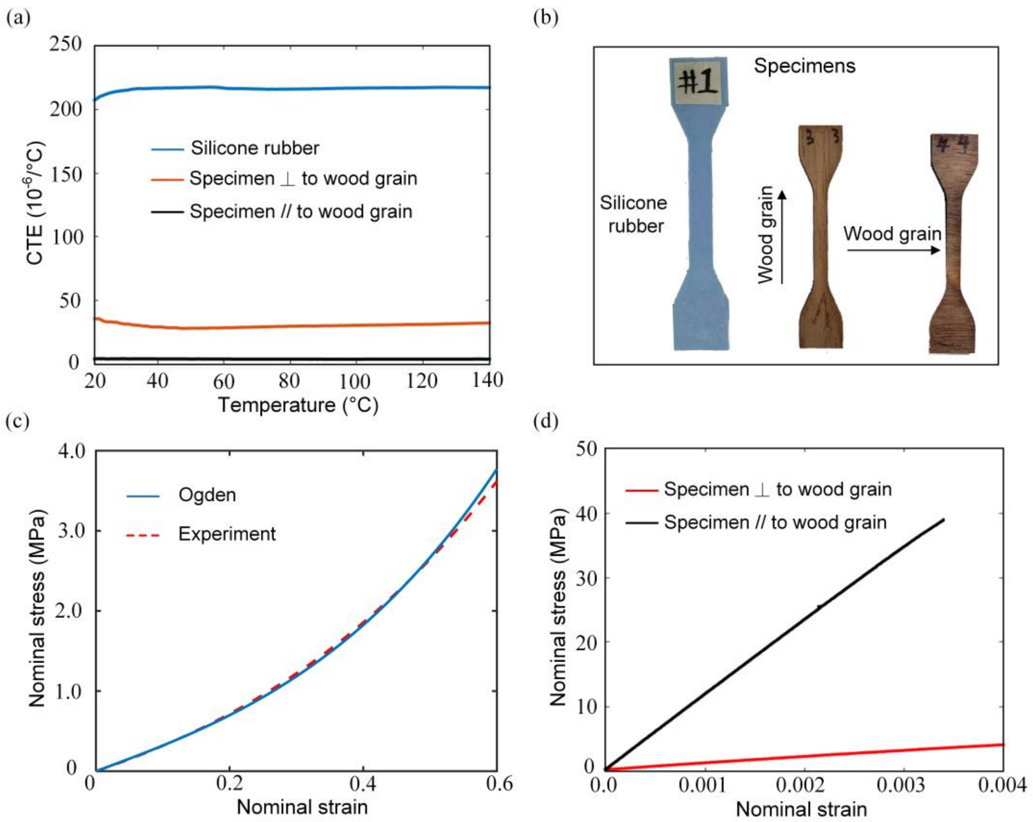

Supplementary Fig. S3: Characterization of material constituents. (a) Experimental measures of the coefficient of thermal expansion for silicone rubber and hardwood. (b) Dumbbell-shaped specimens for uniaxial tensile test. (c) Experimental nominal stress-strain curve of silicone rubber and Ogden fit response. (d) Stress-strain curves obtained from testing hardwood specimens oriented in the direction parallel and perpendicular to the wood grain.

S3. Metaunit analysis: unidirectional and rotational response

S3-A. Theoretical model

This section examines the mechanics of the functional building block shown in Fig.1a subject to a uniform thermal field. We theoretically study the temperature-driven deformation for both the unidirectional and rotational floppy modes. Theoretical expressions for the elastic deflection of both U-BB and R-BB are obtained as a function of temperature, T, under the following assumptions:

- Both constituent solids have constant CTE within the temperature range here investigated.
- Hooke's law of elasticity holds for the high CTE core.
- The low CTE frame is assumed rigid, therefore the deformation induced by the thermal mismatch of the two materials is neglected.
- The length d of the connection between the upper and lower portions of the high CTE core is considered significantly smaller than the BB length ($d \ll l$).

i. Unidirectional Building Block (U-BB)

Supplementary Fig. S4a shows U-BB in its undeformed state: two distinct portions joined centrally via a connection of length d along the horizontal axis of symmetry. Each is shaped with a low CTE (grey) and high CTE (blue) material, where l and width h describe the length and width of the core, which is cut along semi ellipses with major axis of length $2a$ and minor axis of length $2b$. The thermal mismatch between the low CTE confinement (grey) and the high CTE expansion (blue) (Supplementary Fig. S4b) governs the unidirectional response that is endowed by the BB topology. A temperature increase expands the core, which is constricted horizontally by the surrounding frame, and thus forced along its floppy direction to space the upper and lower portion of the BB by Δh, here assumed anchored at its center. Due to the negligible thermal expansion of the low CTE solid, an effective boundary condition is enforced to replace the frame action onto the core. For the sake of symmetry, a quarter of the core is examined with one end clamped and the other free to move transversely via a guided support (Supplementary Fig. S4c). In addition, we simplify the semielliptical quarter of the groove by straightening its profile so as to form an inclined beam, which is in turn attached to the preceding horizontal part. The analysis of the building block subjected to uniform temperature is now reduced to the solution of a statically indeterminate problem of a Timoshenko beam-column (Supplementary Fig. S4d) with an equivalent CTE, $\alpha_{\text{effective}} = \alpha_{\text{core}} - \alpha_{\text{frame}}$, compensating the neglect of the thermal expansion of the frame.

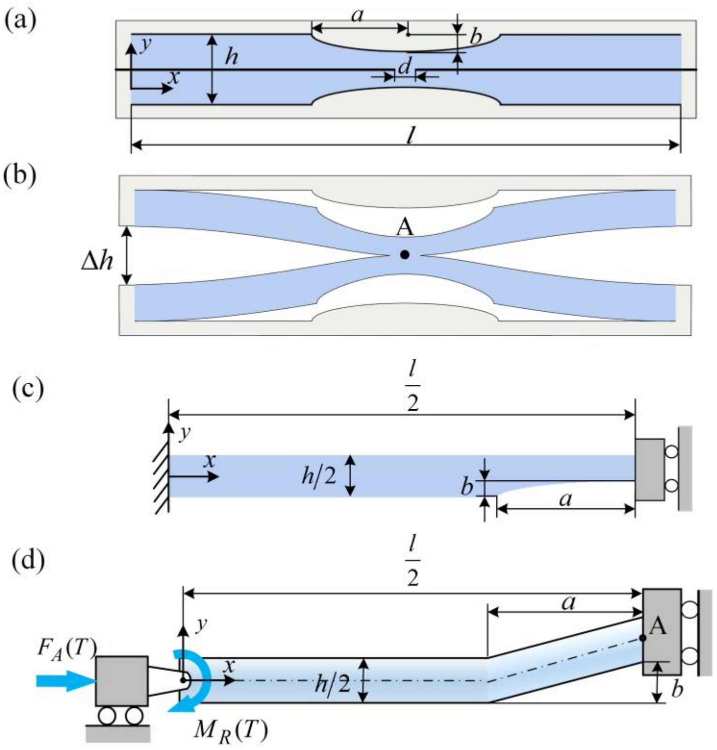

Supplementary Fig. S4: (a) Geometry of unidirectional building block, U-BB, with its dimensional parameters. (b) Deformed U-BB with expansion Δh depicted at a given temperature, T. (c) Condensed model of U-BB, where the nominal geometry is reduced to that of an effective core with $\alpha_{\text{effective}} = \alpha_{\text{core}} - \alpha_{\text{frame}}$ subject to ΔT and pertinent boundary conditions. (d) Further model reduction to a statically determinate Timoshenko beam-column subjected to reaction forces, F_A and M_R, both dependent on T.

By releasing redundant constraints on the clamped end of the beam-column, the model in Supplementary Fig. S4c becomes statically determinate (Supplementary Fig. S4d). Its corresponding axial force ($F_A(T)$) and bending moment ($M_R(T)$) that both depend upon the level of temperature, as well as its deflection can be obtained through the solution of the following set of second-order differential equations:

$$\begin{cases} \dfrac{d^2 v}{dx^2} = -\dfrac{F_A(T)}{EI} v + \dfrac{M_R(T)}{EI}, & 0 \le x \le \left(\dfrac{l}{2} - a\right) \\ \dfrac{d^2 v}{dx^2} = -\dfrac{F_A(T)}{EI} \left[v + \dfrac{b}{a}\left(x - \dfrac{l}{2} + a\right)\right] + \dfrac{M_R(T)}{EI}, & \left(\dfrac{l}{2} - a\right) < x \le \dfrac{l}{2} \end{cases} \tag{2}$$

where v is the transverse deflection along the y axis of the beam, and it represents the difference between the deformed and undeformed axis of the beam-column. E and I are the Young's modulus and the second moment of area of the beam cross-section. The following notation applies:

$$k^2 = \frac{F_A(T)}{EI}, \quad mk^2 = \frac{M_R(T)}{EI}, \quad l_1 = \frac{l}{2} - a \tag{3}$$

which is substituted into Eqns. (2) and yields:

$$\begin{cases} \dfrac{d^2v}{dx^2} = -k^2v + mk^2, & 0 \le x \le l_1 \\ \dfrac{d^2v}{dx^2} = -k^2\left[v + \dfrac{b}{a}(x - l_1)\right] + mk^2, & l_1 < x \le \dfrac{l}{2} \end{cases} \tag{4}$$

The general solutions of Eqns. (4) have the form:

$$\begin{cases} v(x) = C_1 \sin(kx) + C_2 \cos(kx) + m, & 0 \le x \le l_1 \\ v(x) = C_3 \sin(kx) + C_4 \cos(kx) + \dfrac{b}{a}(l_1 - x) + m, & l_1 < x \le \dfrac{l}{2} \end{cases} \tag{5}$$

The constants of integration C_1 and C_2 can be determined from the boundary conditions. Since the beam is clamped at the left end, we impose:

$$v|_{x=0} = 0, \quad \left.\frac{dv}{dx}\right|_{x=0} = 0 \tag{6}$$

To determine the constants of integration C_3 and C_4 , continuity on deflection and slope is enforced at the junction between the two portions of the beam, thus yielding:

$$v|_{x={l_1}^+} = v|_{x={l_1}^-}, \quad \left.\frac{dv}{dx}\right|_{x={l_1}^+} = \left.\frac{dv}{dx}\right|_{x={l_1}^-} \tag{7}$$

Substituting into Eqns. (5) the values of the integration constants from Eqns. (6) and (7) allows the calculation of the deflection curve $v(x)$. By adding the initial ordinates of the undeformed axis to the transverse deflection of the beam, we obtain the final ordinates of the deflection curve for both portions of the beam:

$$\begin{cases} y(x) = -m\cos(kx) + m, & 0 \le x \le l_1 \\ y(x) = -\left[m + \dfrac{b}{ak}\sin(kl_1)\right]\cos(kx) + \dfrac{b}{ak}\cos(kl_1)\sin(kx) + m, & l_1 < x \le \dfrac{l}{2} \end{cases} \tag{8}$$

With the above equations of the deflection curve, we can determine the force F_A and moment M_R by imposing pertinent compatibility conditions. The first specifies that at the right end support of the beam:

$$\left.\frac{dy}{dx}\right|_{x=\frac{l}{2}} = \frac{b}{a} \tag{9}$$

Substitution of Eqns. (8) into Eqn. (9) yields the first compatibility equation:

$$\left[mk + \frac{b}{a}\sin(kl_1)\right]\sin(\frac{kl}{2}) + \frac{b}{a}\cos(kl_1)\cos(\frac{kl}{2}) = \frac{b}{a} \tag{10}$$

The second comes from the observation that upon heating any change in the beam length is caused by the sum of two portions: (1) the elongation produced by the thermal expansion of the beam, and (2) the contraction caused by the axial force acting on the beam. This relation can be expressed as:

$$s(T) - s(T_0) = \Delta s_{\text{expansion}} + \Delta s_{\text{compression}} \tag{11}$$

where $s(T_0)$ and $s(T)$ are the length of the beam axis at room temperature T_0 and at a higher temperature T, respectively. $\Delta s_{\text{thermal}}$ and $\Delta s_{\text{compression}}$ represent the length change of the beam caused by thermal expansion and axial compression, respectively. The expressions are given by:

$$\begin{aligned} s(T) - s(T_0) &= \int_0^{l_1} (ds - dx) + \int_{l_1}^{\frac{l}{2}} \left(ds - dx\sqrt{1 + (b/a)^2} \right) \\ \Delta s_{\text{thermal}} &= \alpha_{\text{effective}} (T - T_0) \left(l_1 + a\sqrt{1 + (b/a)^2} \right) \\ \Delta s_{\text{compression}} &= -\frac{F_A l}{2EA} \end{aligned} \quad (12)$$

Substitution of Eqns. (12) into Eqn. (11) results in the second compatibility equation:

$$\frac{1}{8}\left[k^2m^2l + \frac{2b^2}{a} + \frac{2mb}{a}\cos(kl_1) + 4mkb\sin(kl_1) - km^2\sin(kl) - \frac{2mb}{a}\cos(kl - kl_1) + \frac{b^2}{a^2k}\sin(2ka) \right] + a\left(1 - \sqrt{1 + (b/a)^2}\right) + \frac{F_A l}{2EA} - \alpha_{\text{effective}}(T - T_0)\left(l_1 + a\sqrt{1 + (b/a)^2} \right) = 0 \quad (13)$$

Since a compact closed-form for $F_A(T)$ and $M_R(T)$ cannot be directly retrieved from the above equations, we numerically solve Eqns. (13) and (10) and use the Newton–Raphson method to find their roots. The deflection curve $y_T(x)$ of the beam at a given temperature T can then be obtained by substituting the roots of $F_A(T)$ and $M_R(T)$ into Eqns. (8). The expansion of the building block Δh at a given temperature T is thus determined by calculating the transverse displacement of the beam at point A, expressed by:

$$\Delta h(T) = 2\left[y_T\left(\frac{l}{2}\right) - b \right] \quad (14)$$

ii.Deformation mode of U-BB

The above theory can now be used to investigate the deformation mechanism of U-BB for an increasing level of temperature. To do so, we examine a representative beam-column with given parameters l/h =9, $2a/l$ =0.1, and $2b/h$ =0.2 and plot the reaction and bending moment at the beam end. Supplementary Fig. S5a shows the results with the axial force F_A and bending moment M_R plotted as a function of the temperature range here investigated. To rule out size dependency in the plot, F_A and M_R are normalized with the cross-sectional dimensions $h/2$ and t_t, the latter representing the out-of-plane thickness of BB. Two sequential regimes of deformation can be observed, each controlled by temperature. For low values of temperature, the axial force dominates the bending moment and increases linearly with temperature; here axial compression governs the U-BB response. With a further increase of temperature, the deformation mode switches. Bending increases steeply and non-linearly, and the axial force flattens at a plateau with a resulting bending domination at high temperature. This is also apparent in Supplementary Fig. S5b, where a transition from a stretching to a bending mode appears in an intermediate zone (grey) around T=60 °C. Below this zone, i.e. in the low temperature regime, the change in slope of $\Delta h/h$ shows a moderate

expansion induced by extension. In contrast above the transition zone, U-BB expansion is controlled by internal bending of the core with a rate of $\Delta h/h$ that increase swiftly and non-linearly with temperature.

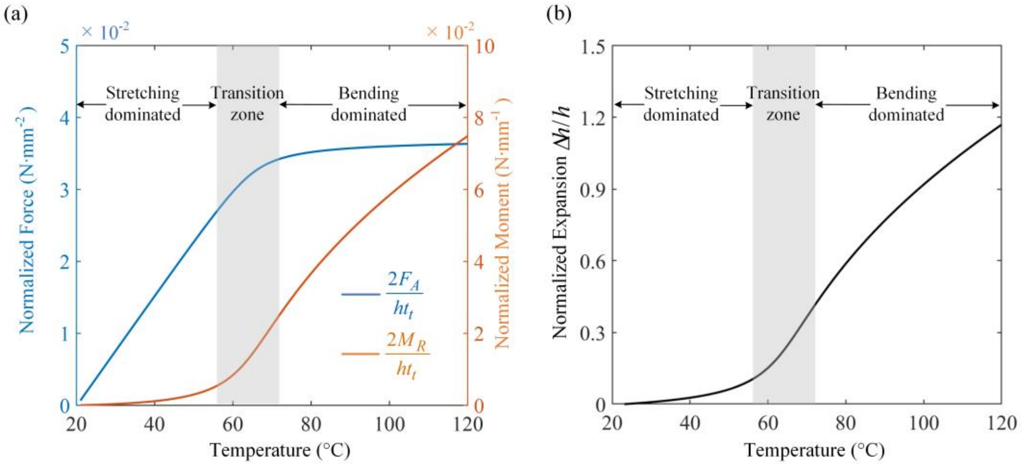

Supplementary Fig. S5: (a) Evolution of normalized F_A and M_R as a function of temperature in the temperature range 20 to 120 °C. (b) Normalized expansion $\Delta h/h$ as a function of temperature with slope showing BB sensitivity to temperature variation in the low and high temperature regime, respectively below and above the transition zone.

iii.Rotational Building Block (R-BB)

R-BB differs from U-BB for a mere symmetry breaking as well as end closure of the core. They both seal R-BB with a rotational character. Supplementary Fig. S6a shows a shift in the position of the connection d of the core at an offset e from the left end of R-BB. The underlying mechanism of thermal deformation does not differ from that of U-BB, i.e. the trigger is the CTE mismatch of the constituents, but the topological difference between the two (U versus R) is responsible for each floppy mode (Supplementary Fig. S6b).

To study the theory governing R-BB mechanics, we amend the beam-column model previously examined for U-BB in Supplementary Fig. S4. Deformation symmetry in R-BB occurring along the dash-dot axis (Supplementary Fig. S6b) enables us to focus on the lower half of the high CTE core only, this time with both ends clamped. The reaction from the upper part is equivalent to a bending moment ($M_S(T)$) applied at A, i.e. the core connection (Supplementary Fig. S6c). By releasing redundant constraints on the clamped end (Supplementary Fig. S6d), we obtain three reaction forces $F_A(T)$, $F_V(T)$, and $M_R(T)$ with values dependent on temperature. As opposed to the U-BB analysis, this time a transverse force F_V appears to balance M_S.

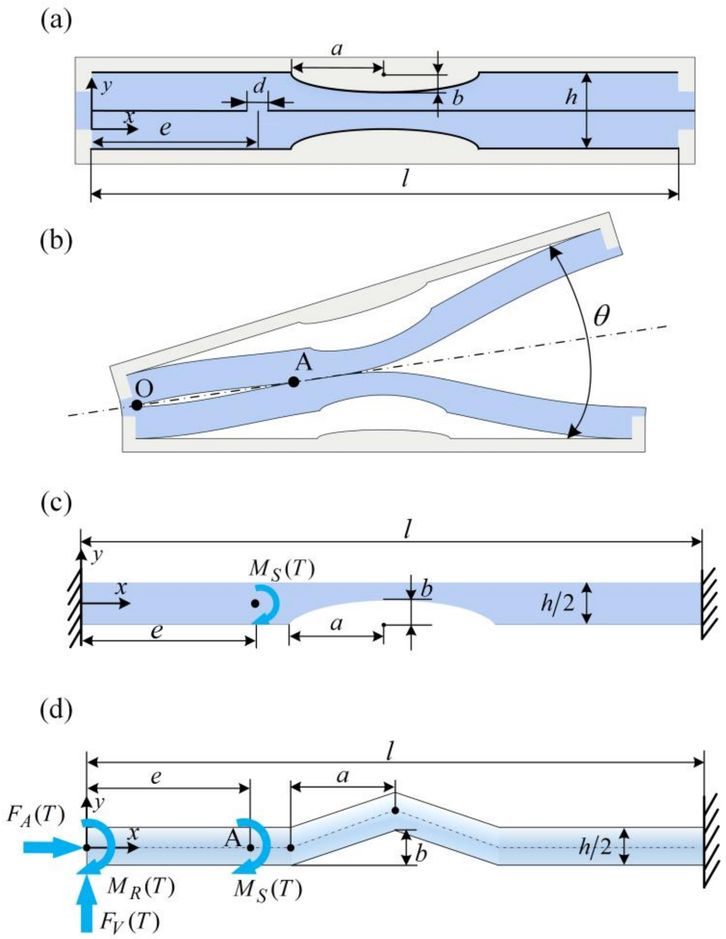

Supplementary Fig. S6: (a) Geometry of rotational building block, R-BB. (b) Deformed R-BB with opening angle θ shown at a given T. (c) Reduced model for BB analysis, where the R-BB is simplified to the high CTE core subject to T and pertinent boundary conditions. (d) The high CTE core model is here condensed to a statically determinate Timoshenko beam-column with reaction and internal forces F_A, F_V, M_R and M_S, all dependent on temperature.

Supplementary Fig. S6d shows the four portions of the Timoshenko beam-column representing R-BB. Similar to the U-BB analysis of the preceding section, also here we obtain the ordinates of the deflection curve for the five segments of the polygonal beam:

$$\begin{cases} y(x) = C_1 \sin(kx) + C_2 \cos(kx) + m + nx, & 0 \leq x < e \\ y(x) = C_3 \sin(kx) + C_4 \cos(kx) + m + p + nx, & e \leq x < l_1 \\ y(x) = C_5 \sin(kx) + C_6 \cos(kx) + m + p + nx, & l_1 \leq x < l/2 \\ y(x) = C_7 \sin(kx) + C_8 \cos(kx) + m + p + nx, & l/2 \leq x < l_2 \\ y(x) = C_9 \sin(kx) + C_{10} \cos(kx) + m + p + nx\ , & l_2 \leq x \leq l \end{cases} \quad (15)$$

where

$$k^2 = \frac{F_A}{EI}, \quad mk^2 = \frac{M_R}{EI}, \quad nk^2 = \frac{F_V}{EI}, \quad pk^2 = \frac{M_S}{EI}, \quad l_1 = \frac{l}{2} - a, \quad l_2 = \frac{l}{2} + a \quad (16)$$

The constants of integration $C_1, C_2, \ldots, C_{10}$ are determined from the boundary conditions and continuity at the junction between neighbouring portions of the beam:

$$
\begin{aligned}
&C_1 = -\frac{n}{k}, && C_2 = -m,\\
&C_3 = C_1 - p\sin(ke), && C_4 = C_2 - p\cos(ke),\\
&C_5 = C_3 + \frac{b}{ak}\cos(kl_1), && C_6 = C_4 - \frac{b}{ak}\sin(kl_1),\\
&C_7 = C_5 - \frac{2b}{ak}\cos\left(\frac{kl}{2}\right), && C_8 = C_6 + \frac{2b}{ak}\sin\left(\frac{kl}{2}\right),\\
&C_9 = C_7 + \frac{b}{ak}\cos(kl_2), && C_{10} = C_8 - \frac{b}{ak}\sin(kl_2),
\end{aligned} \tag{17}
$$

The reactions forces (F_A, F_V, M_R, M_S) are now obtained by imposing four pertinent compatibility conditions. First, Eqn. (11), which for U-BB retains beam length compatibility upon heating, still holds for R-BB. Hence, substituting the expression of $s(T) - s(T_0)$, $\Delta s_{\text{thermal}}$, and $\Delta s_{\text{compression}}$ into Eqn. (11) results in:

$$
\begin{gathered}
\int_0^{l_1}(ds - dx) + \int_{l_1}^{l_2}\left(ds - dx\sqrt{1 + r^2}\right) + \int_{l_2}^{l}(ds - dx) + \frac{F_A l}{EA}\\
-\alpha_{\text{effective}}(T - T_0)\left(l - 2a + 2a\sqrt{1 + (b/a)^2}\right) = 0
\end{gathered} \tag{18}
$$

In addition, the following two apply to the right end support of the beam:

$$y(l) = 0 \tag{19}$$

$$\left.\frac{dy}{dx}\right|_{x=l} = 0 \tag{20}$$

The fourth condition stems from the deformation symmetry along the OA line that exists in the deformed R-BB (Supplementary Fig. S6b). This translates into an equivalent condition of slope between point A (offset of e from O), and the symmetry axis (OA), here expressed as:

$$\left.\frac{dy}{dx}\right|_{x=e} = \frac{y(e)}{e} \tag{21}$$

Also in this case, we resort to the Newton–Raphson method to numerically solve the reaction force F_A, F_V, M_R, M_S from the system of Eqns. (18)-(21). After the deflection curve y_T of the beam at a given temperature T is obtained by substituting their roots into Eqns. (15), we can express the opening angle, θ, of R-BB at a given temperature T through the slope of point A on the deflection curve as:

$$\theta(T) = 2\arctan\left(\left.\frac{dy_T}{dx}\right|_{x=e}\right) \tag{22}$$

iv.Deformation mode of R-BB

Similar to the U-BB investigation on the mechanism of deformation, here we study the deformation behaviour of R-BB. As a representative model, a beam-column is examined with l/h =9, $2a/l$ =0.1, $2b/h$ =0.2, $2e/l$ =0.8. Supplementary Fig. S7a and b plot its reaction and internal forces F_A, F_V, M_R, and M_S as a function of increasing temperature. The trends (Supplementary Fig. S7a) are similar to those of U-BB:

two temperature dependent modes with deformation switch from stretching to bending occurring above the transition zone (grey). In Supplementary Fig. S7b the magnitude of F_V and M_S are respectively two and one order lower than F_A and M_R in Supplementary Fig. S7a. This result indicates that the transverse force and bending moment at the connection (point A in Supplementary Fig. S6d) contribute only slightly to the R-BB deformation. Furthermore, Supplementary Fig. S7c shows the temperature driven response of R-BB, where the slope of the opening angle (θ) indicates a mild deformation dominated by extension in the low temperature regime, as opposed to above, where BB responds with internal bending with a deformation that is rapid and sensitive to the temperature change at higher values.

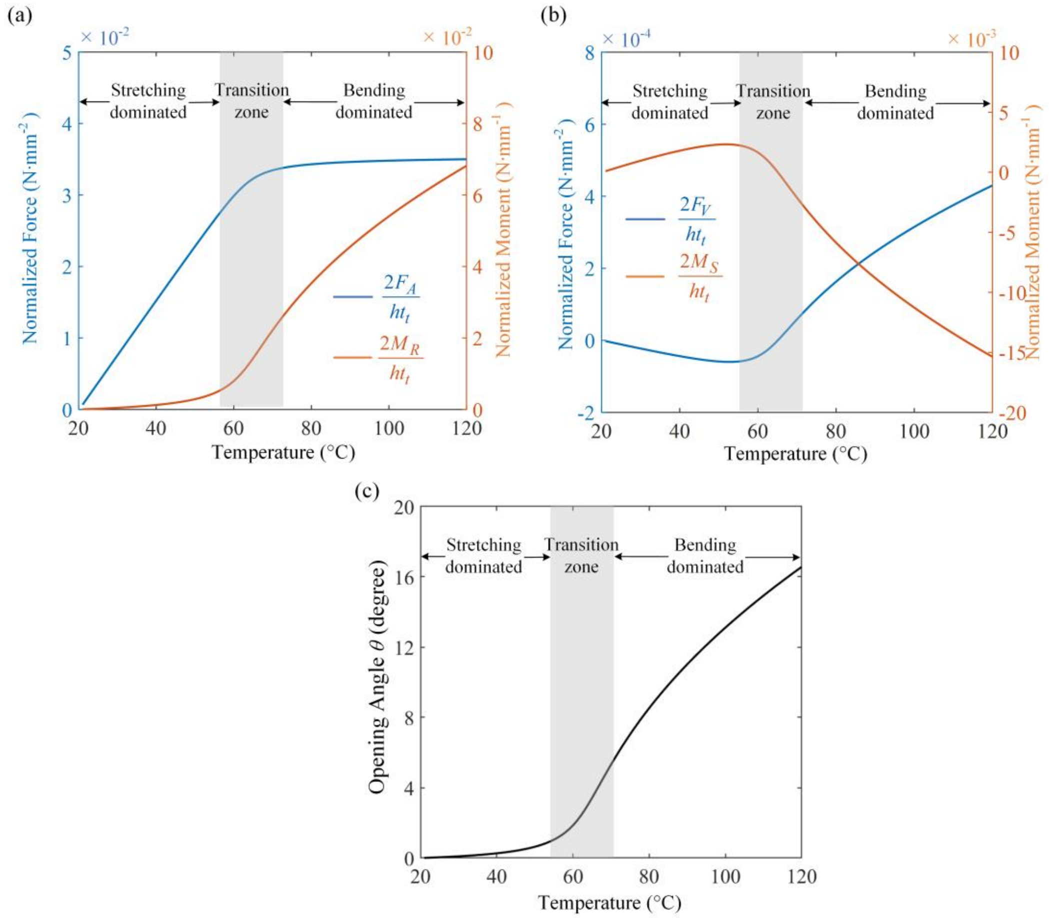

Supplementary Fig. S7: (a) and (b) Evolution of the normalized F_A, M_R, and F_V, M_S as a function of temperature in the range 20 to 120 °C. (c) Opening angle θ as a function of temperature with slope showing R-BB sensitivity to a temperature change throughout the whole spectrum of temperature.

S3-B. Experimental validation of BB theoretical models

To verify the validity of the solutions obtained from the previous theoretical analyses, we fabricated a number of building blocks with a specific set of geometric parameters and tested them in a heating chamber

(see S1 for details). A representative array of results from these experiments is shown in Supplementary Fig. S8 for both U-BB and R-BB. The experimentally measured values of $\Delta h/h$ and θ of actual BB geometry confirms the mechanism of deformations predicted through the mechanics theory on simplified column-beam analogs. In Supplementary Fig. S8, the experimental results show a good agreement with the theoretical prediction, especially in high temperature region (max error 15%). The discrepancy can be attributed to frictional dissipation and the simplified geometry assumed for the BB. In the low temperature regime, the former plays a major role, since the testing plate upon which the BB rests during the experiment is not perfectly smooth and hence the frictional force can significantly dominate the low magnitude of the internal forces generated in BB (Supplementary Fig. S7a and b). In the high temperature regime, on the other hand, the simplification of the semielliptical groove of BB core to a straight inclined beam is the main source of the deviation.

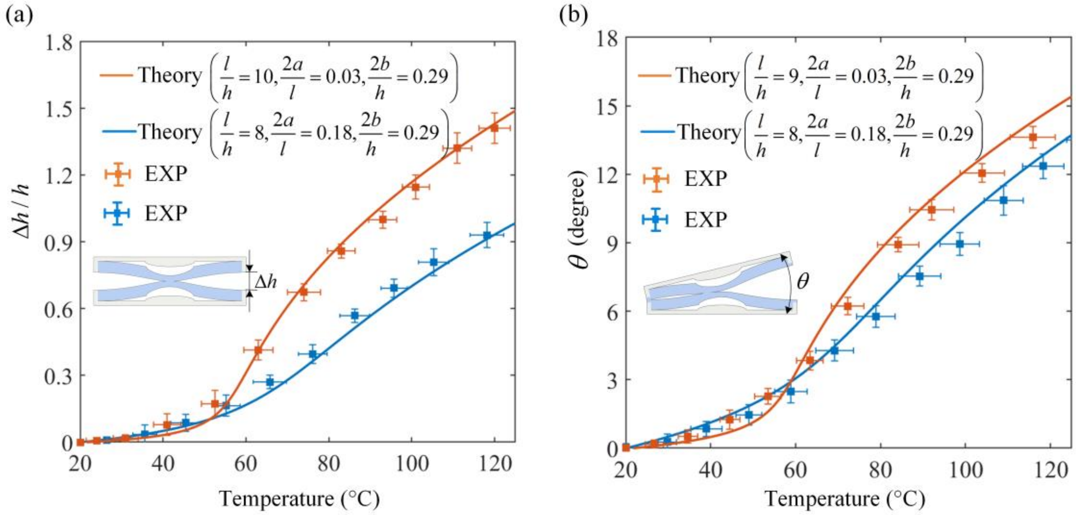

Supplementary Fig. S8: Experimental validation of theoretical predictions for both BBs: (a) normalized expansion $\Delta h/h$ versus temperature for U-BB tested with two representative sets of parameters (blue and red); (b) opening angle θ as a function of temperature for R-BB tested with two representative sets of parameters. Horizontal and vertical bars indicate the standard deviation around the mean from a pool of measures taken for both temperature and deformation, the former measured at three distinct sites in the heating camber, the latter obtained from three repeated measurements.

S3-C. Computational analysis of BB response

The theory presented in S3-A and experimentally validated in S3-B applies to a BB with core of small size ligament (*d*<<*l*) and groove geometry abridged with a straight axis. Here we relax these assumptions and examine a BB with actual (un-simplified) core geometry made of two distinct passive materials: silicone rubber and wood (see S2 for material properties). Section S3-C-i explains the method used to generate the deformation property capturing the role of the ligament size (*d/l*) besides the core size (*h/l*). Section S3-C-ii investigates the minor role of other geometric parameters defining U-BB and R-BB, in particular, the groove size, the core connection offset and the size of the end closure.

All the computational analyses here performed account for both material and geometric non-linearity of the constituents and are conducted by combining Python scripts and Abaqus (Dassault Systemes Simulia Corp, France). Eight-node, quadratic plane-stress elements (element type CPS8) are used to discretize the BB models and a mesh size equal to $b/4$ is adopted after performing a convergence analysis on the mesh size. The mechanical behavior of silicone rubber core is captured by a first order Ogden model. The effective properties of the wooden frame are assumed as transversely isotropic, and the values are obtained from experimental measures on dog-bone specimen of the solid material (see S2 for material properties). The cut motifs introduced into the computational models are represented with seam cracks along which duplicate element nodes overlap. A contact law with hard contact for the normal behavior, and frictionless for the tangential behavior, was assigned to the model.

i. Deformation-property profile

The deformation property profile in Fig.1a is generated through a response surface that fits a set of numerical predictions with model details given above. In general, the relationship between the response of a system (y) and a set of predictor variables ($x_1, x_2, \dots, x_k$) can be mathematically expressed as a multiple linear regression model which can be written as

$$y = \beta_0 + \beta_1 x_1 + \beta_2 x_2 + \cdots + \beta_k x_k + \varepsilon \quad (23)$$

where y is the true response variable and the parameters β_j $(j = 0,1, \dots, k)$, are the regression coefficients. This model spans a k-dimensional space defined by the regressor $\{x_j\}$. The parameter ε is the error of the regression model.

To provide a continuous approximation of the true response, the material and geometry spaces of the deformation-property profile of BB are generated from two sets of simulations. These are conducted in the admissible domain of two sets of variables: $(E_f/E_c, \Delta\alpha)$ and $(l/h, d/l)$, and for each of them a response surface is obtained. In general, the response of an *N*-order model as a function of two variables, x_1 and x_2, is given by:

$$y = \sum_{I}^{N} \sum_{J=0}^{I} \beta_{I,J} x_1^J x_2^{I-J} + \varepsilon \tag{24}$$

where y is the true value of the response, in this case, $\Delta h/h$ for U-BB and θ for R-BB; x_1 and x_2 are E_f/E_c, $\Delta\alpha$ for the material space, and l/h, d/l for the geometric space. If we let $\beta_{I,J} = \beta_{(I^2+3I-2J)/2}$, and $x_1^J x_2^{I-J} = x_{(I^2+3I-2J)/2}$, where indices I and J are integers satisfying $0 \le I + J \le N$ and $J \le I$, we can write Eqn. (24) in the general form of a multiple linear regression model as in Eqn. (23).

To estimate the regression coefficients β_j, we use the method of the least squares. Writing Eqn. (23) in matrix notation gives:

$$\boldsymbol{y} = \boldsymbol{X\beta} + \boldsymbol{\varepsilon} \tag{25}$$

where:

$$\boldsymbol{y} = \begin{bmatrix} y_1 \\ y_2 \\ \vdots \\ y_n \end{bmatrix}, \boldsymbol{X} = \begin{bmatrix} 1 & x_{11} & x_{12} & \cdots & x_{1k} \\ 1 & x_{21} & x_{22} & \cdots & x_{2k} \\ \vdots & \vdots & & \vdots & \\ 1 & x_{n1} & x_{n2} & \cdots & x_{nk} \end{bmatrix}, \boldsymbol{\beta} = \begin{bmatrix} \beta_1 \\ \beta_2 \\ \vdots \\ \beta_k \end{bmatrix}, and\ \boldsymbol{\varepsilon} = \begin{bmatrix} \varepsilon_1 \\ \varepsilon_2 \\ \vdots \\ \varepsilon_n \end{bmatrix} \tag{26}$$

where the index *n* represents the number of sampling points in the design of experiments.

Since the goal is to find the regression coefficient vector $\boldsymbol{\beta}$ that minimizes the error vector $\boldsymbol{\varepsilon}$, we can write the set of least squares function as:

$$L = \sum_{i=1}^{n} \varepsilon_i^2 = \boldsymbol{\varepsilon}^{\mathrm{T}}\boldsymbol{\varepsilon} = (\boldsymbol{y} - \boldsymbol{X\beta})^{\mathrm{T}}(\boldsymbol{y} - \boldsymbol{X\beta}) \tag{27}$$

Eqn. (27) can be further developed into:

$$L = \boldsymbol{y}^{\mathrm{T}}\boldsymbol{y} - \boldsymbol{2\beta}^{\mathrm{T}}\boldsymbol{X}^{\mathrm{T}}\boldsymbol{y} + \boldsymbol{\beta}^{\mathrm{T}}\boldsymbol{X}^{\mathrm{T}}\boldsymbol{X\beta} \tag{28}$$

with the least-squares estimators $b_0, b_1, \dots, b_k$ satisfying the condition:

$$\left.\frac{\partial L}{\partial \boldsymbol{\beta}}\right|_{\boldsymbol{b}} = -2\boldsymbol{X}^{\mathrm{T}}\boldsymbol{y} + \boldsymbol{2X}^{\mathrm{T}}\boldsymbol{Xb} = \boldsymbol{0} \tag{29}$$

Eqn. (29) simplifies to the normal equations in matrix form:

$$\boldsymbol{X}^{\mathrm{T}}\boldsymbol{Xb} = \boldsymbol{X}^{\mathrm{T}}\boldsymbol{y} \tag{30}$$

Solving the normal equations gives the least-squares estimator $\boldsymbol{b}$ of the regression coefficients $\boldsymbol{\beta}$:

$$\boldsymbol{b} = \left(\boldsymbol{X}^{\mathrm{T}}\boldsymbol{X}\right)^{-1}\boldsymbol{X}^{\mathrm{T}}\boldsymbol{y} \tag{31}$$

Hence, the fitted regression model is:

$$\hat{\boldsymbol{y}} = \boldsymbol{Xb} \tag{32}$$

The residuals are:

$$\boldsymbol{e} = \boldsymbol{y} - \hat{\boldsymbol{y}} \tag{33}$$

The coefficient of determination is:

$$R^2 = \frac{SS_R}{SS_T} = 1 - \frac{SS_E}{SS_T} \tag{34}$$

From the above we can respectively write the sum of squares of the regression SS_R, the sum of squares of the residual SS_E, and the total sum of squares as:

$$SS_R = \boldsymbol{b}^{\mathrm{T}}\boldsymbol{X}^{\mathrm{T}}\boldsymbol{y} - \frac{(\sum_{i=1}^{n} y_i)}{n} \tag{35}$$

$$SS_E = \boldsymbol{e}^{\mathrm{T}}\boldsymbol{e} = \boldsymbol{y}^{\mathrm{T}}\boldsymbol{y} - \boldsymbol{b}^{\mathrm{T}}\boldsymbol{X}^{\mathrm{T}}\boldsymbol{y} \tag{36}$$

$$SS_T = SS_R + SS_E \tag{37}$$

To reduce the error of the approximation, we aim at ensuring the coefficient of determination, R^2, above 0.99, a value that indicates here an acceptable level of accuracy. As per choice of the order of the response surface function (Eqn. (24)), we adopt $N = 4$ after performing a systematic analysis on the role of N.

While Fig.1a shows the response surface of $\Delta h/h$ for U-BB, i.e. the property-deformation profile, here we report, as example, the expression of the surface response of θ for R-BB in its geometric space:

$$\theta = \sum_{I}^{4} \sum_{J=0}^{I} c_{I,J} \left(\frac{l}{h}\right)^{J} \left(\frac{d}{l}\right)^{I-J} \tag{38}$$

where the regression coefficients $c_{I,J}$ is estimated through Eqn. (31). A similar approximation can be expressed in the complementary material space for R-BB as a function of the pair of material properties $(E_f/E_c, \Delta\alpha)$. Below are two plots constituting the property-deformation profile of R-BB response at $T = 120$ °C (Supplementary Fig. S9a and b). Obtained through Eqn. (38), the spectra provide the range of rotational deformation that a BB can achieve through tuning the most influential geometric and material attributes that control R-BB response to temperature. The maps provide guidelines for attributes selection of BB, in particular, the geometric space on the right-hand side is the foundation of our morphing scheme.

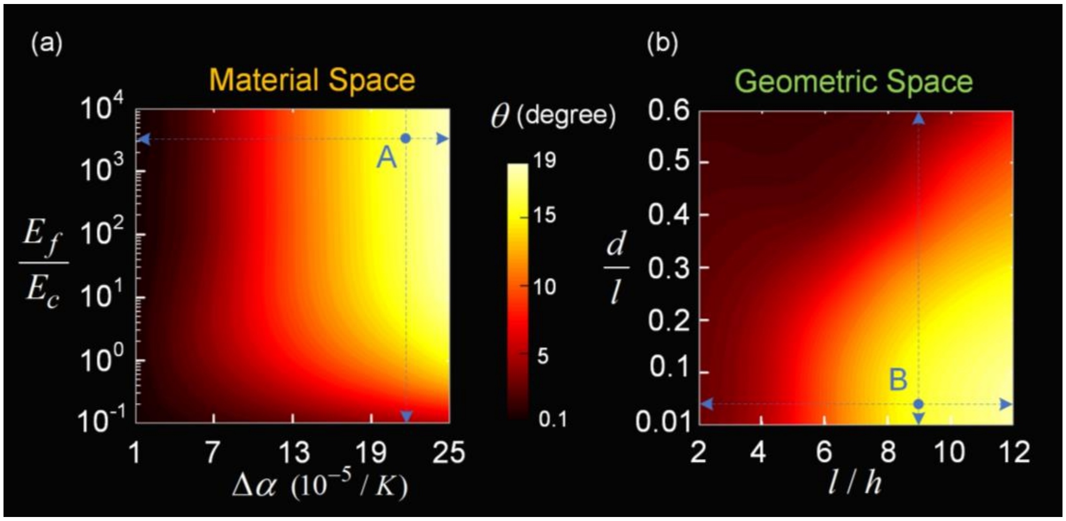

Supplementary Fig. S9: Property-deformation profile of R-BB

ii. Sensitivity to least influential geometric parameters

a. Role of groove size (a/l and b/h) in U-BB

While the surface response in Fig.1 shows the role of the most influential geometric parameters, l/h and d/l, on $\Delta h/h$, Supplementary Fig. S10 reports complementary results capturing the influence of the semielliptical groove of the core: ($2a/l$) and ($2b/h$). For a BB with l/h equal to 9, the curves show a minor role of $2a/l$ and $2b/h$ in the high temperature regime. On the other hand, in the transition zone, i.e. mid-range temperature, more notable differences appear in the shape of the response curve. In particular, for low values of $2a/l$ and $2b/h$, e.g. 0.02, the curves resemble those obtained through theory with the appearance of two regimes of deformation. On the other hand, for larger size of the groove, e.g. $2a/l$=0.4 or $2b/h$=0.4, no transition zone can be observed; here U-BB steadily expands with temperature through a combination of bending and stretching generated in its members.

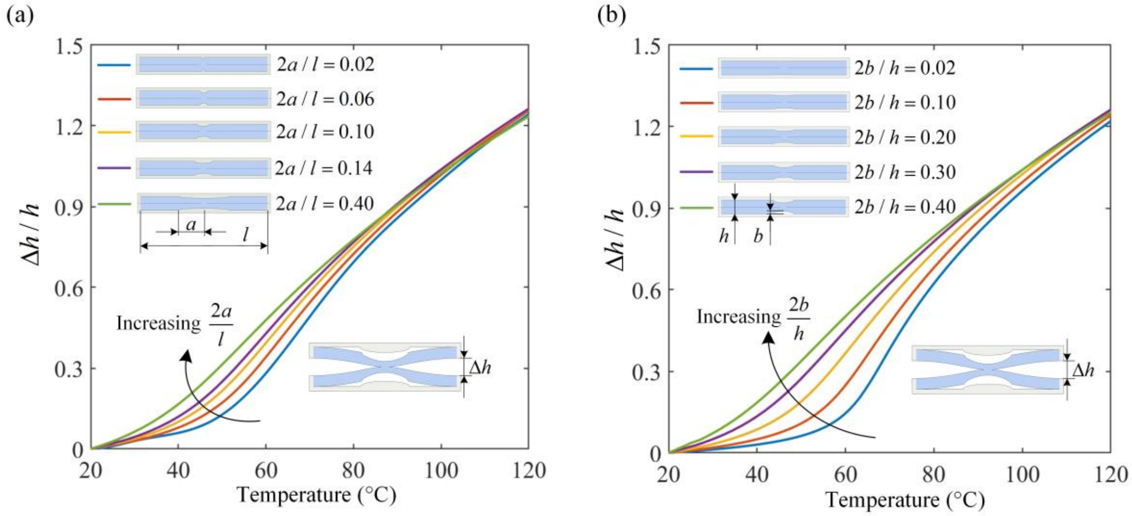

Supplementary Fig. S10: Normalized expansion of U-BB for a set of varying parameters of the semielliptical groove: (a) major axis: $2a/l$=0.02, 0.06, 0.1, 0.14, 0.4; (b) minor axis $2b/h$ =0.02, 0.1, 0.2, 0.3, 0.4.

b. Role of core connection offset, and end closure size in R-BB

Similarly, Supplementary Fig. S11a and b show θ for varying values of ($2a/l$) and ($2b/h$), for a BB with l/h equal to 9. In addition, for R-BB, we investigate the role of the connection offset, e, and the size of the end closure n.

Supplementary Fig. S11c and d show that the opening angle increases with the $2e/l$ and n/h. Compared to l/h or d/l, however, their effect is minor, and thus the tunability they would offer to tailor R-BB

response is narrow. To maximize BB response tunability, the values of the BB parameters chosen in this work are: $2a/l = 10/63$, $2b/l = 2/7$, $2e/l = 4/5$, $n/h = 2/7$.

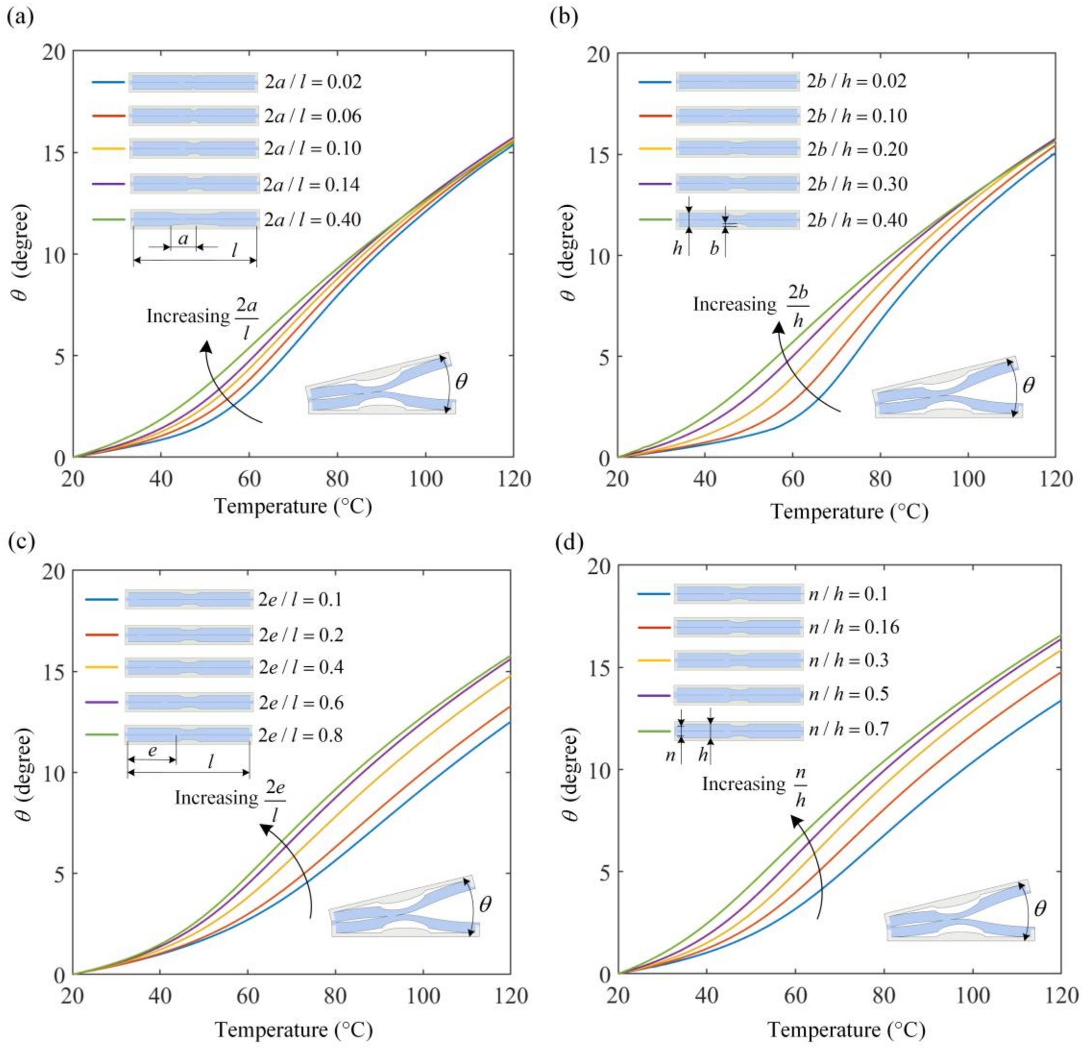

Supplementary Fig. S11: Opening angle θ of R-BB for a set of varying parameters: (a) major axis: $2a/l$=0.02, 0.06, 0.1, 0.14, 0.4; (b) minor axis $2b/h$ =0.02, 0.1, 0.2, 0.3, 0.4; (c) $2e/l$=0.1, 0.2, 0.4, 0.6, 0.8; (d) n/h=0.1, 0.16, 0.3, 0.5, 0.7.

S4. Investigation on stress state and bond strength of silicone-wood interface

Supplementary Fig. S12a shows that the constituent materials after the curing process bond at four sites, thus creating four silicone/wood interfaces. It is at these locations that the BB can potentially become weak during deformation. Here we analyze the stress state of their interfaces resulting from an increase of temperature and then measure the bonding strength through a set of pull-out tests.

Section S3-A provides a full analysis of both the temperature-driven deformation and the internal forces generated in a given BB. The results show the existence of an axial compressive force F_R and a bending moment M_R that act on the bonding interface (Supplementary Fig. S12a). At each of these edges, the distribution of the normal stresses can be simply described as:

$$\sigma(y, T) = -\frac{M_R(T)}{I}y - \frac{F_R(T)}{A} \tag{39}$$

where σ is the normal stress exerted by the core to the surrounding low CTE frame, $y \in [-h/4,\ h/4]$, and T is a given temperature. A positive value of σ represents a tensile stress, whereas the negative counterpart indicates compression. A and I are the area and second moment of area of the cross-section. From Eqn. (39), we gather that the lower half of the cross-section ($y \in [-h/4, 0]$) is subject to either compressive or tensile stress, depending on the magnitude of M_R and F_R, which is governed by temperature. Only the tensile portion can lead to interfacial debonding, which is controlled by the maximum tensile stress that occurs at the furthest edge ($y = -h/4$) of the cross-section. This critical value varies with the BB core size. Supplementary Fig. S12b-II shows how the normal stress at $y = -h/4$ evolves with temperature and the aspect ratio (l/h) of the BB core. The green area indicates a negative value of $\sigma(y = -h/4)$, meaning the transversal section of the interface is entirely compressed (Supplementary Fig. S12b-I). On the other hand, the orange points to a regime with positive values, which correspond to the distribution of $\sigma(y = -h/4)$ shown in Supplementary Fig. S12b-III. Only in this regime, debonding may occur, once the normal tensile stress at $y = -h/4$ reaches a critical value above the strength of the bonded interface.

To determine the strength of the bonding interface, we conducted a set of uniaxial tensile tests on six specimens fabricated with the process described in Fig.2. Specimens were extracted from a representative sample with geometry emphasizing the wood/silicone interface, i.e. a critical portion of one unit with two beams of rubber bonded to a layer of wood (Supplementary Fig. S12c-I). The bonding strength was assessed through a set of pull-out tests (Supplementary Fig. S12c-II). The tensile force was applied until the appearance of interfacial debonding, a point identified in red in the force-displacement curve of Supplementary Fig. S12c-IV. From the value of the ultimate force at this point, the bonding strength was calculated as 0.65 ± 0.09 MPa, a value that is an order of magnitude higher than the critical values that tensile stress can assume in the tensile regime (orange in Supplementary Fig. S12b-II). From this investigation, we can assert that the stress state at the silicone-wood interface is either compression or tension depending on temperature. While the former cannot but contribute to amplify the bonding of silicone rubber to wood, the latter can reach only values that are much lower than the bonding strength, hence providing almost no likelihood of detachment under the conditions and temperature reported in this investigation.

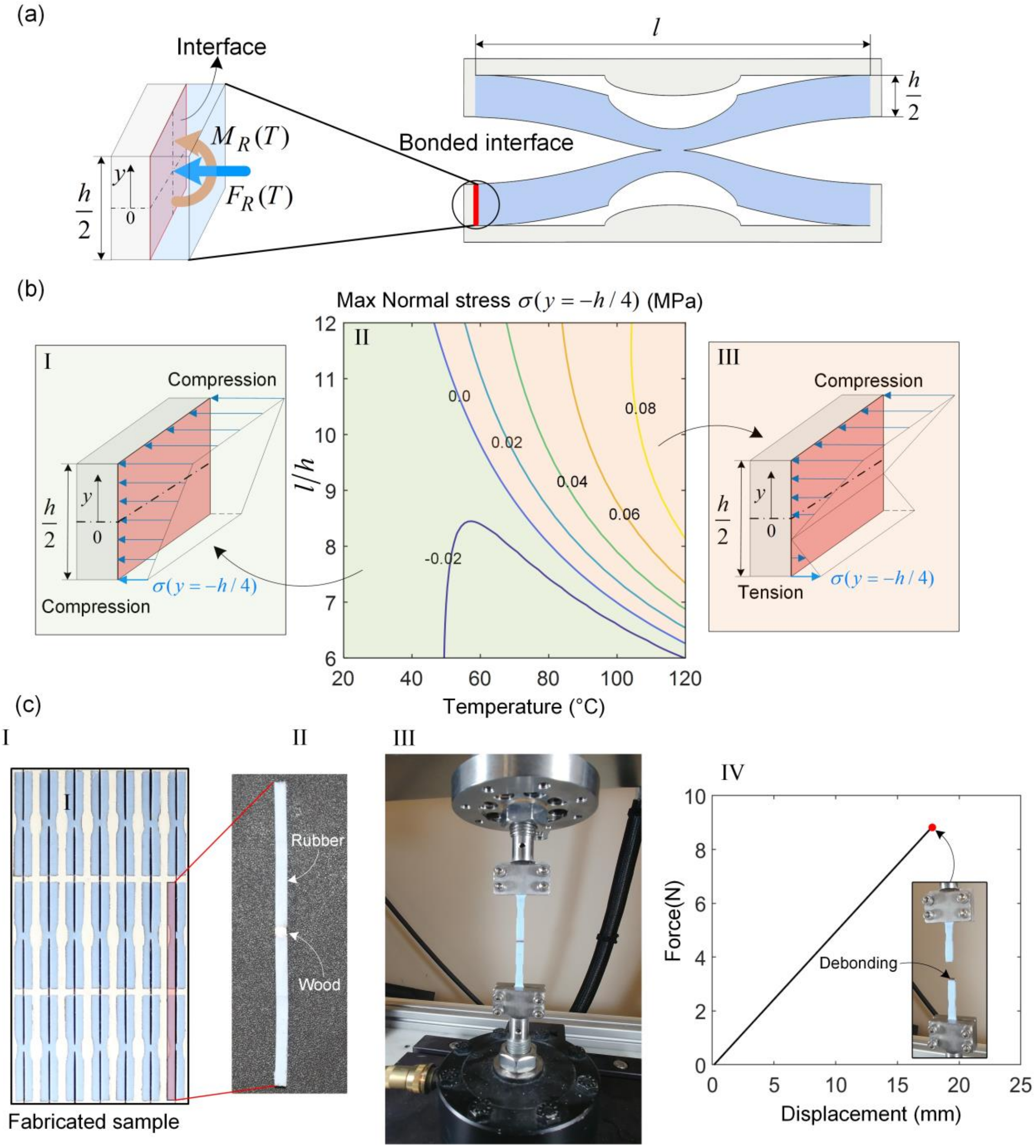

Supplementary Fig. S12: (a) Internal forces triggered by an increase of temperature at the bonding interface sites of BB. (b) Normal stress σ at $y = -h/4$ as a function of temperature T and length-to-width ratio of the BB core l/h. (c) Pull-out test and force-displacement response of a representative specimen.

S5. Time response upon heating

As described in S3-A, the deformation of the BB is caused by a mere mismatch of thermal expansion of the constituent materials. The materials do not undergo any atomic or molecular changes, as in the case of smart materials, such as shape memory polymers. On the other hand, the deformation of our BB is determined by the topological layout and the internal forces that are generated in the BB members, with magnitude dependent on temperature. Once the BB has reached the target temperature, the deformation is instantaneous, as opposed to the time response observed in smart materials. With our metamaterials, the only time span involved is that required to heat the sample to the target temperature, which depends only on the heating strategy and the experimental setup.

Our analysis here, therefore, pertains to our experimental system (see S1), which consisted of a heating chamber with multiple thermocouples monitoring the maintenance of 120 ℃, our target temperature. We conducted a series of tests on a BB placed in the middle of the chamber already heated at 120 ℃ and we recorded with a digital camera (EOS Rebel T6i, Canon USA) the time span required for the BB to deform. Two medium types were examined: fan-propelled air and oil bath, the former requiring 300±17 seconds to complete the deformation, and the latter 115±18 seconds.

To corroborate our experimental observations, a numerical analysis of the transient heat was performed with the following values assigned to the thermal conductivity, K, and specific heat, c, of the constituents: $K_{rubber} = 0.2\,W/(m \cdot K)$, $c_{rubber} = 1100\,J/(kg \cdot K)$, and $K_{wood} = 0.1\,W/(m \cdot K)$, $c_{wood} = 1600\,J/(kg \cdot K)$. Due to symmetry, half BB was modelled in three dimensions (Supplementary Fig. S13a) with size identical to the fabricated BB and discretized with 20-node quadratic heat transfer bricks (DC3D20 element) in Abaqus (Dassault Systemes Simulia Corp, France). The following convective conditions were applied to the external surfaces of the model:

$$q = h_c(T_f - T) \tag{40}$$

where q and h_c are respectively the heat flux per second and the heat convection coefficient. T denotes the surface temperature, with initial value set to 20 ℃, and T_f represents the temperature of the surrounding medium, assumed here as 120 ℃. Because the heat convection coefficient h_c cannot be accurately extracted from our in-house heating chamber, we appraised the upper and lower bound of the time span that would result from values of the heat convection h_c falling within practical ranges. For natural convection in oil, the h_c range was 50 - 200 $\mathrm{W}/(m^2K)$, and for the fan-propelled air heating (force convection with low speed air over surface), it was 10 - 40 $\mathrm{W}/(m^2K)$.

Supplementary Fig. S13b and c show the time variation of the interior temperature within the silicone rubber core for a BB immersed respectively in air and oil at 120 ℃. A rapid increase can be observed until the thermal equilibrium is reached, a state indicated by the start of the plateau with corresponding time

representing the BB response. In air, the response is within the range 180 to 660 seconds, whereas in oil it is 56 to 160 seconds. These ranges are aligned with the values obtained from the experiments.

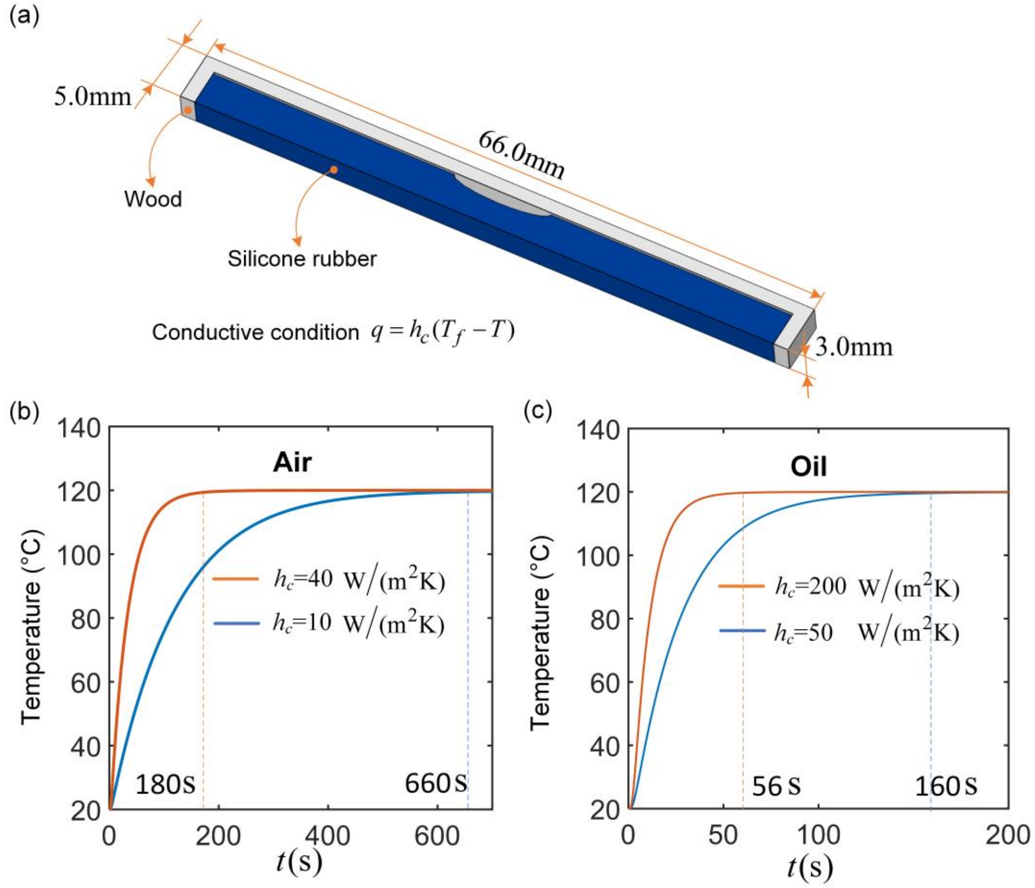

Supplementary Fig. S13: (a) Numeric results from transient heat transfer. (b) Interior temperature of BB as a function of time for BB in fan-propelled air conditions. (c) Interior temperature of BB as a function of time for BB immersed in oil bath.

S6. Morphing from a pre-assigned sequence of building blocks

S3 has examined the response of an individual BB through theory and numerical analysis. Here we focus on a monolithic assembly of BBs, each with its own set of geometric attributes. The goal is to express the collective deformation of the metamaterial phenotype at a given T. As a paradigmatic example, we consider a series of m rotational units sharing low CTE edges and all having equal width l_i. Supplementary Fig. S14 shows its deformed configuration, the phenotype at a given T. The central curve (dash type) is specified as representative of the deflected axis, and assumed as an arc spline consisting of a number of G^1 continuous arcs and straight segments, all passing through the interface mid-points, P_i, between adjacent units and normal to their contiguous edges.

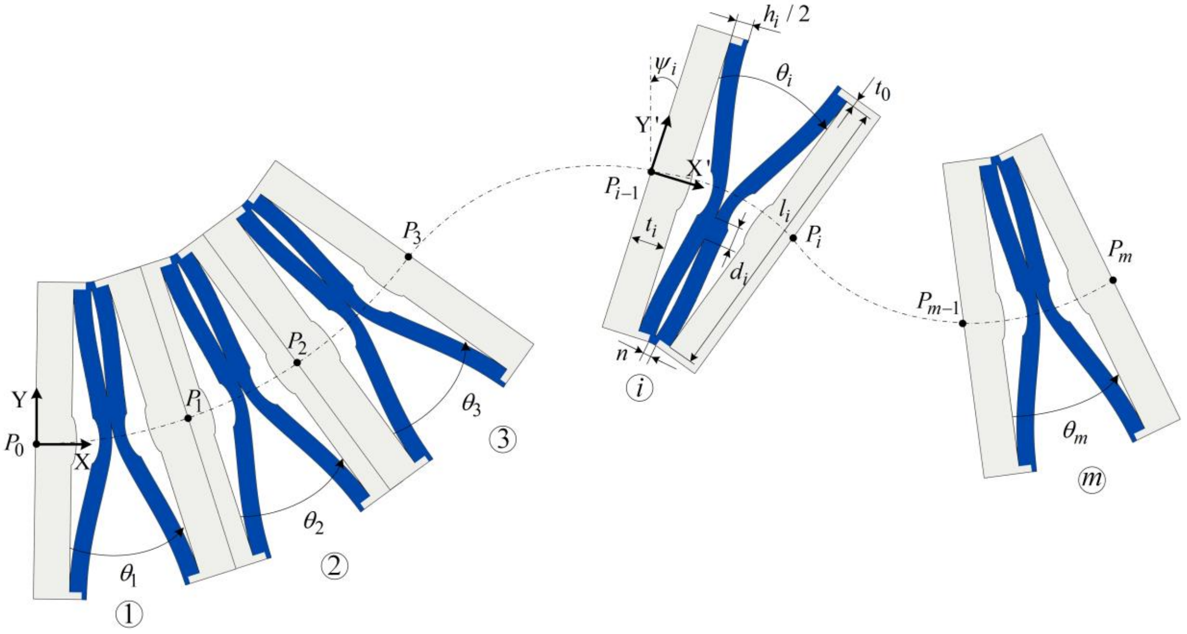

Supplementary Fig. S14: Schematic of a chain of m building blocks in series, with an arc connecting a pair of consecutive mid-points P_i at the BB interface. The local coordinate system (X′Y′) of a generic unit i is referenced to the global system (XY) on the first outer frame of the first unit.

With reference to the generic building block i, we express the coordinates of its point P_i in the local coordinate system, indicated as the X′Y′ plane, and in the global system residing at P_0 and visualized as the XY plane in the first unit. The two systems are rotated by ψ_i and translated by a vector pointing from point P_o to P_{i-1}. The coordinates of P_i in the X′Y′ plane can be represented by the two-dimensional array:

$$\boldsymbol{p}'_i = \begin{bmatrix} x'_{p_i} \\ y'_{p_i} \end{bmatrix} = \begin{bmatrix} \cos(\theta_i/2)L_i \\ -\sin(\theta_i/2)L_i \end{bmatrix} \tag{41}$$

where the integer $i \in [1, m]$ and the expression of L_i, which is the distance between two consecutive mid-points of the low CTE edges, is given by:

$$L_i = (h_i - n + 2t_i)\cos(\theta_i/2) + (l_i + 2t_0)\sin(|\theta_i|/2) + n \quad (42)$$

where h_i, l_i, t_i, d_i are the main geometric parameters controlling the R-BB response, and n, t_0 are minor parameters here assumed unchanging for all the units due to their minor influence (Supplementary Fig. S11). θ_i represents the opening angle of building block i, whose value is governed by l_i/h_i and d_i/l_i, as illustrated in (Supplementary Fig. S9b). A positive value of θ_i represents a clockwise rotation, and negative means counter-clockwise.

The following affine transformation can be used to express P_i in the global coordinate system (XY):

$$\boldsymbol{p}_i = \boldsymbol{M}_i \boldsymbol{p'}_i + \boldsymbol{v}_i \quad (43)$$

where the array $\boldsymbol{p}_i$ represents the coordinates of P_i in the global coordinates system. $\boldsymbol{v}_i$ denotes the translation vector pointing from point P_o to P_{i-1}; the matrix $\boldsymbol{M}_i$ defines the relative rotation of the coordinate systems by the angle ψ_i, and are given by:

$$\boldsymbol{M}_i = \begin{bmatrix} \cos\psi_i & -\sin\psi_i \\ \sin\psi_i & \cos\psi_i \end{bmatrix}, \qquad \boldsymbol{v}_i = \boldsymbol{p}_{i-1} - \boldsymbol{p}_0 \quad (44)$$

If the origin of the global coordinate system is P_0 with coordinates $x_{p_0} = y_{p_0} = 0$, the rotation of the unit i with respect to the global system (XY) is the mere aggregation of the opening angle θ of the preceding units $(i-1)$ (see Supplementary Fig. S14), thus expressed as

$$\psi_i = \sum_{j=0}^{i-1} \theta_j \quad (45)$$

where θ_0 is null. As assumed above, the central axis of the deformed configuration of the BB assembly can be represented by a G^1 continuous arc spline passing through all the mid-points P_i. The expression of a generic arc i in the arc spline can be expressed from point P_{i-1} and to point P_i, as:

$$F_i(x, y) = (x - A_i)^2 + (y - B_i) - R_i^2 = 0\,, \qquad \begin{matrix} x_{p_{i-1}} \le x \le x_{p_i} \\ y_{p_{i-1}} \le y \le y_{p_i} \end{matrix} \quad (46)$$

with R_i being the radius, and (A_i, B_i) the coordinates of the arc center, whose expressions are:

$$R_i = \frac{L_i}{2\sin(|\theta_i|/2)}, \quad A_i = -\frac{L_i \sin\psi_i}{2\sin(\theta_i/2)} + x_{p_{i-1}}, \quad B_i = -\frac{L_i \cos\psi_i}{2\sin(\theta_i/2)} + y_{p_{i-1}} \quad (47)$$

S7. Morphing on-target through encoded sequence of building blocks

Morphing on target poses the search of a BB sequence that allows the metamaterial phenotype to accurately conform to a target domain. We describe here the target as a domain with central axis representable with an arc spline and two symmetric boundaries that are continuous with varying distance along the central axis. Both are shown in blue on the upper part of Supplementary Fig. S15, the former in dash-dot and the latter with solid line. In red, at the lower part of the figure, is a metamaterial phenotype with randomly assigned BB sequence expressed at a given temperature.

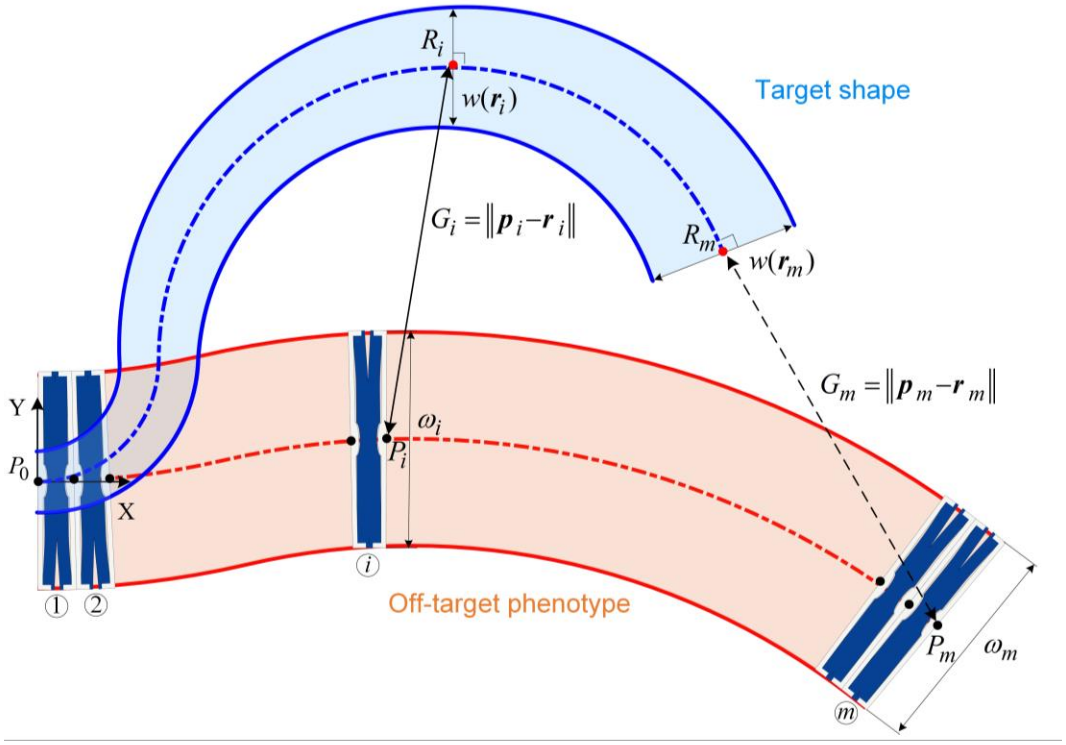

Supplementary Fig. S15: Target domain (blue - top) with varying width and off-target metamaterial phenotype (red - below), both sharing the initial location where the global coordinate system is anchored.

S7-A. Description of target shape

The central axis of the target shape is here assumed to trace an arc spline mathematically expressed piece-wisely in an implicit form as:

$$f = f(x,y) = \begin{cases} f_1(x,y) = 0, & \text{if } 0 \le x < x_1 \quad 0 \le y < y_1 \\ f_2(x,y) = 0, & \text{if } x_1 \le x < x_2 \quad y_1 \le y < y_2 \\ \quad \cdots & \\ f_q(x,y) = 0, & \text{if } x_{q-1} \le x \le x_q \quad y_{q-1} \le y \le y_q \end{cases} \tag{48}$$

where q denotes the number of arcs and straight lines, and the intervals are the coordinates of the blending points.

For the symmetric boundaries of the target shape, the function describing the width change along the central axis is expressed as:

$$w = w(x, y) \tag{49}$$

S7-B. Problem formulation

The phenotype will match the target shape if two conditions are guaranteed. The first pertains to the central axis of the metamaterial phenotype. For a given temperature, a generic phenotype with randomly assigned BB sequence will most likely be off-target, i.e. its central axis would appear far from that of the target. This gap from the target (Supplementary Fig. S15) can be mathematically expressed for each building block as

$$G_i = \sqrt{\left[(x_{p_i} - x_{r_i})^2 + (y_{p_i} - y_{r_i})^2\right]} = \|\boldsymbol{p_i} - \boldsymbol{r_i}\| \tag{50}$$

where (x_{p_i}, y_{p_i}) are the coordinates of the interface mid-point P_i of BB i in the off-target phenotype, and (x_{r_i}, y_{r_i}) are those of the respective point R_i on the target axis; $\boldsymbol{p_i}$ and $\boldsymbol{r_i} = \left[x_{r_i}, y_{r_i}\right]^{\mathrm{T}}$ denote the position vectors of P_i and R_i in the global coordinate system.

The second condition relates to the boundary of the target domain. Again here, an arbitrary string of BBs will likely lead to a phenotype with non-conforming boundaries, i.e. incompatible from those of the target (Supplementary Fig. S15). We thus express the gap between the width of each BB and the width of the boundary at a given position as:

$$W_i = \left|\omega_i - w(x_{r_i},\ y_{r_i})\right| = |\omega_i - w(\boldsymbol{r_i})| \tag{51}$$

where $\omega_i = l_i + 2t_0$ is the width for BB_i in the off-target phenotype, and $w(x_{r_i},\ y_{r_i})$ is the width of the target shape corresponding to point R_i.

In general, the characteristic distances of an off-target phenotype from the target domain, i.e. Eqns. (50) and (51), should be both minimized simultaneously. This can be expressed through a least-squares model that minimizes the sum of the squares of both residuals. In this case, the overall objective function can be formulated as:

$$F = \sum_{i=1}^{m}\left(G_i^2 + W_i^2\right) = \sum_{i=1}^{m}[\|\boldsymbol{p_i} - \boldsymbol{r_i}\|^2 + |\omega_i - w(\boldsymbol{r_i})|^2] \tag{52}$$

S7-C. Design variables

Supplementary Fig. S14 shows the internal parameters describing the BB architecture: $h_i, t_i, l_i, d_i, n, t_0$. The first four are treated as variables, whereas n and t_0 are assumed constant because (as S3-C

demonstrates) the BB response is less sensitive to changes in their value. We can thus collect the most influential descriptors of the BB geometry in the following vector of variables:

$$\boldsymbol{X} = \{h_i, t_i, l_i, d_i\}^{\mathrm{T}} \quad (i = 1,2,..m) \tag{53}$$

S7-D. Design constraints

To achieve the kinematic compatibility between neighbouring units, the edge of each building block i should be normal to the tangent direction of the target axis, an arc spline, on point R_i. These constraints can be mathematically expressed by the system of equalities:

$$g_i = \boldsymbol{n}_i^{\mathrm{T}} \cdot \boldsymbol{u}_i = 0 \quad (i = 1,2,\ldots,m) \tag{54}$$

where $\boldsymbol{n}_i$ denotes the normal vector of the target curve on point R_i, expressed as:

$$\boldsymbol{n}_i = \left[\partial f\left(x_{r_i}, y_{r_i}\right)/\partial x,\ \partial f\left(x_{r_i}, y_{r_i}\right)/\partial y\right]^{\mathrm{T}} \tag{55}$$

with $\partial f/\partial x$ and $\partial f/\partial y$ representing partial derivatives of the target arc spline; (x_{r_i}, y_{r_i}) denote the coordinates of point R_i in the global coordinate system. $\boldsymbol{u}_i$ is a vector in the X axis direction of the localized coordinate system residing at point P_{i-1}, and it can be obtained from the following transformation:

$$\boldsymbol{u}_i = \boldsymbol{M}_i \boldsymbol{u}_i' \tag{56}$$

The unit vector $\boldsymbol{u_i'}$ is expressed as $[1,0]^{\mathrm{T}}$ in the local coordinate system and the matrix $\boldsymbol{M}_i$ **(**Eqn. (44)) defines the relative rotation.

The opening angle θ_i for each R-BB is governed by both l_i/h_i and d_i/l_i, and Supplementary Fig. S9b presents the maximum range of θ_i that R-BB can generate in the geometric space. To produce admissible deformation, the following constraints should not be violated:

$$\underline{g}_i^{l/h} = R_{l/h}^L - l_i/h_i \leq 0 \tag{57}$$

$$\overline{g}_i^{l/h} = l_i/h_i - R_{l/h}^U \leq 0 \tag{58}$$

$$\underline{g}_i^{d/l} = R_{d/l}^L - d_i/l_i \leq 0 \tag{59}$$

$$\overline{g}_i^{d/l} = d_i/l_i - R_{d/l}^U \leq 0 \tag{60}$$

where $R_{l/h}^L$ and $R_{l/h}^U$ are the lower and upper bounds on the ratio of l_i/h_i, respectively, and $R_{d/l}^L$ and $R_{d/l}^U$ are lower and upper bounds on d_i/l_i.

For practical considerations, side constraints are also set for each design variable, i.e. $h_i^L \leq h_i \leq h_i^U, t_i^L \leq t_i \leq t_i^U, l_i^L \leq l_i \leq l_i^U, d_i^L \leq d_i \leq d_i^U$, where the superscripts L and U represent the lower and upper bounds on the design variables, respectively.

S7-E. Optimization problem formulation

The problem of morphing on-target is thus expressed as the minimization of the sum of the squares of both gaps (Eqns. (50) and (51)) subject to the following set of equality and inequality constraints:

$$\begin{cases} \min\limits_{h_i,t_i,l_i,d_i} \quad F = \sum_{i=1}^{m}[\|\boldsymbol{p_i} - \boldsymbol{r_i}\|^2 + |\omega_i - w(\boldsymbol{r_i})|^2] \\ \text{s.t.} \qquad g_i = 0, \\ \underline{g}_i^{l/h} \le 0, \\ \overline{g}_i^{l/h} \le 0, \\ \underline{g}_i^{d/l} \le 0, \\ \overline{g}_i^{d/l} \le 0, \\ h_i^L \le h_i \le h_i^U, \\ t_i^L \le t_i \le t_i^U, \\ l_i^L \le l_i \le l_i^U, \\ d_i^L \le d_i \le d_i^U, \qquad i = 1,2,\dots,m. \end{cases} \tag{61}$$

S7-F. Sensitivity analysis

The derivatives of the objective function with respect to the design variables h_j, t_j, $l_j$and d_j ($j = 1,2,\dots,m$) can be obtained through

$$\frac{\partial F}{\partial h_j} = \sum_{i=1}^{m}\left[\left(\frac{\partial \boldsymbol{p_i}}{\partial h_j}\right)^{\mathrm{T}}(\boldsymbol{p_i} - \boldsymbol{r_i}) + (\boldsymbol{p_i} - \boldsymbol{r_i})^{\mathrm{T}}\left(\frac{\partial \boldsymbol{p_i}}{\partial h_j}\right)\right] \tag{62.a}$$

$$\frac{\partial F}{\partial t_j} = \sum_{i=1}^{m}\left[\left(\frac{\partial \boldsymbol{p_i}}{\partial t_j}\right)^{\mathrm{T}}(\boldsymbol{p_i} - \boldsymbol{r_i}) + (\boldsymbol{p_i} - \boldsymbol{r_i})^{\mathrm{T}}\left(\frac{\partial \boldsymbol{p_i}}{\partial t_j}\right)\right] \tag{62.b}$$

$$\frac{\partial F}{\partial l_j} = \sum_{i=1}^{m}\left[\left(\frac{\partial \boldsymbol{p_i}}{\partial l_j}\right)^{\mathrm{T}}(\boldsymbol{p_i} - \boldsymbol{r_i}) + (\boldsymbol{p_i} - \boldsymbol{r_i})^{\mathrm{T}}\left(\frac{\partial \boldsymbol{p_i}}{\partial l_j}\right)\right] + 2\left(\omega_j - w(\boldsymbol{r_j})\right) \tag{62.c}$$

$$\frac{\partial F}{\partial d_j} = \sum_{i=1}^{m}\left[\left(\frac{\partial \boldsymbol{p_i}}{\partial d_j}\right)^{\mathrm{T}}(\boldsymbol{p_i} - \boldsymbol{r_i}) + (\boldsymbol{p_i} - \boldsymbol{r_i})^{\mathrm{T}}\left(\frac{\partial \boldsymbol{p_i}}{\partial d_j}\right)\right] \tag{62.d}$$

The partial derivatives of Eqn. (62) are

$$\frac{\partial \boldsymbol{p_i}}{\partial h_j} = \begin{cases} \sum_{k=j+1}^{i}\frac{\partial \theta_j}{\partial h_j}\begin{bmatrix} -sin(\psi_k + \theta_k/2)L_k \\ cos(\psi_k + \theta_k/2)L_k \end{bmatrix} + \boldsymbol{M}_j\frac{\partial \boldsymbol{p}'_j}{\partial h_j}, & i > j \\ \boldsymbol{M}_j\frac{\partial \boldsymbol{p}'_j}{\partial h_j}, & i = j \\ \boldsymbol{0}, & i < j \end{cases} \tag{63.a}$$

$$\frac{\partial \boldsymbol{p_i}}{\partial t_j} = \begin{cases} \boldsymbol{M}_j\frac{\partial \boldsymbol{p}'_j}{\partial t_j}, & i \ge j \\ \boldsymbol{0}, & i < j \end{cases} \tag{63.b}$$

$$\frac{\partial \boldsymbol{p_i}}{\partial l_j} = \begin{cases} \sum_{k=j+1}^{i} \frac{\partial \theta_j}{\partial l_j} \begin{bmatrix} -sin(\psi_k + \theta_k/2)L_k \\ cos(\psi_k + \theta_k/2)L_k \end{bmatrix} + \boldsymbol{M}_j \frac{\partial \boldsymbol{p}'_j}{\partial l_j}, & i > j \\ \boldsymbol{M}_j \frac{\partial \boldsymbol{p}'_j}{\partial l_j}, & i = j \\ \boldsymbol{0}, & i < j \end{cases} \tag{63.c}$$

$$\frac{\partial \boldsymbol{p_i}}{\partial d_j} = \begin{cases} \sum_{k=j+1}^{i} \frac{\partial \theta_j}{\partial d_j} \begin{bmatrix} -sin(\psi_k + \theta_k/2)L_k \\ cos(\psi_k + \theta_k/2)L_k \end{bmatrix} + \boldsymbol{M}_j \frac{\partial \boldsymbol{p}'_j}{\partial d_j}, & i > j \\ \boldsymbol{M}_j \frac{\partial \boldsymbol{p}'_j}{\partial d_j}, & i = j \\ \boldsymbol{0}, & i < j \end{cases} \tag{63.d}$$

where

$$\boldsymbol{M}_j \frac{\partial \boldsymbol{p}'_j}{\partial h_j} = \frac{1}{2} \begin{bmatrix} cos\psi_{j+1} & cos\psi_j & -sin\psi_{j+1} & -sin(\psi_j + \theta_j/2) \\ sin\psi_{j+1} & sin\psi_j & cos\psi_{j+1} & cos(\psi_j + \theta_j/2) \end{bmatrix} \begin{bmatrix} 1 + sgn(\theta_j)\omega_j \frac{\partial \theta_j}{\partial h_j} \\ 1 \\ (h_j - n + 2t_j) \frac{\partial \theta_j}{\partial h_j} \\ n \frac{\partial \theta_j}{\partial h_j} \end{bmatrix} \tag{64.a}$$

$$\boldsymbol{M}_j \frac{\partial \boldsymbol{p}'_j}{\partial t_j} = \begin{bmatrix} cos\psi_j + cos\psi_{j+1} \\ sin\psi_j + sin\psi_{j+1} \end{bmatrix} \tag{64.b}$$

$$\boldsymbol{M}_j \frac{\partial \boldsymbol{p}'_j}{\partial l_j} = \frac{1}{2} \begin{bmatrix} cos\psi_{j+1} & -sin\psi_j & -sin\psi_{j+1} & -sin(\psi_j + \theta_j/2) \\ sin\psi_{j+1} & cos\psi_j & cos\psi_{j+1} & cos(\psi_j + \theta_j/2) \end{bmatrix} \begin{bmatrix} sgn(\theta_j)\omega_j \frac{\partial \theta_j}{\partial l_j} \\ sgn(\theta_j) \\ (h_j - n + 2t_j) \frac{\partial \theta_j}{\partial l_j} - sgn(\theta_j) \\ n \frac{\partial \theta_j}{\partial l_j} \end{bmatrix} \tag{64.c}$$

$$\boldsymbol{M}_j \frac{\partial \boldsymbol{p}'_j}{\partial d_j} = \frac{1}{2} \frac{\partial \theta_j}{\partial d_j} \begin{bmatrix} cos\psi_{j+1} & -sin\psi_j & -sin(\psi_j + \theta_j/2) \\ sin\psi_{j+1} & cos\psi_j & cos(\psi_j + \theta_j/2) \end{bmatrix} \begin{bmatrix} sgn(\theta_j)\omega_j \\ (h_j - n + 2t_j) \\ n \end{bmatrix} \tag{64.d}$$

$$\frac{\partial \theta_j}{\partial h_j} = \sum_I^N \sum_{J=0}^I - \frac{J c_{I,J}}{h_j} \left(\frac{l_j}{h_j}\right)^J \left(\frac{d_j}{l_j}\right)^{I-J} \tag{64.e}$$

$$\frac{\partial \theta_j}{\partial l_j} = \sum_I^N \sum_{J=0}^I \frac{(2J-I) c_{I,J}}{l_j} \left(\frac{l_j}{h_j}\right)^J \left(\frac{d_j}{l_j}\right)^{I-J} \tag{64.f}$$

$$\frac{\partial \theta_j}{\partial d_j} = \sum_I^N \sum_{J=0}^I \frac{(I-J) c_{I,J}}{l_j} \left(\frac{l_j}{h_j}\right)^J \left(\frac{d_j}{l_j}\right)^{I-J-1} \tag{64.g}$$

The derivatives of the constraint in Eqn. (54) with respect to the design variables h_j, t_j, $l_j$and d_j ($j = 1,2,\ldots,m$) are given by

$$\frac{\partial g_i}{\partial h_j} = \begin{cases} \frac{\partial \theta_j}{\partial h_j}\left(-\frac{\partial f\left(x_{r_i}, y_{r_i}\right)}{\partial x} sin\psi_i + \frac{\partial f\left(x_{r_i}, y_{r_i}\right)}{\partial y} cos\psi_i\right), & i > j \\ 0, & i \leq j \end{cases} \quad (65.a)$$

$$\frac{\partial g_i}{\partial t_j} = 0 \quad (65.b)$$

$$\frac{\partial g_i}{\partial l_j} = \begin{cases} \frac{\partial \theta_j}{\partial l_j}\left(-\frac{\partial f\left(x_{r_i}, y_{r_i}\right)}{\partial x} sin\psi_i + \frac{\partial f\left(x_{r_i}, y_{r_i}\right)}{\partial y} cos\psi_i\right), & i > j \\ 0, & i \leq j \end{cases} \quad (65.c)$$

$$\frac{\partial g_i}{\partial d_j} = \begin{cases} \frac{\partial \theta_j}{\partial d_j}\left(-\frac{\partial f\left(x_{r_i}, y_{r_i}\right)}{\partial x} sin\psi_i + \frac{\partial f\left(x_{r_i}, y_{r_i}\right)}{\partial y} cos\psi_i\right), & i > j \\ 0, & i \leq j \end{cases} \quad (65.d)$$

where $\partial\theta_j/\partial h_j$, $\partial\theta_j/\partial l_j$ and $\partial\theta_j/\partial d_j$ are expressed in Eqns. (64.e), (64.f) and (64.g), respectively.

The derivatives of the constraints in Eqns. (57)-(60) are

$$\frac{\partial \underline{g}_i^{l/h}}{\partial h_j} = \begin{cases} l_j/h_j^2, & i = j \\ 0, & i \neq j \end{cases} \qquad \frac{\partial \underline{g}_i^{l/h}}{\partial l_j} = \begin{cases} -1/h_j, & i = j \\ 0, & i \neq j \end{cases} \qquad \frac{\partial \underline{g}_i^{l/h}}{\partial t_j} = \frac{\partial \underline{g}_i^{l/h}}{\partial d_j} = 0 \quad (66)$$

$$\frac{\partial \overline{g}_i^{l/h}}{\partial h_j} = \begin{cases} -l_j/h_j^2, & i = j \\ 0, & i \neq j \end{cases} \qquad \frac{\partial \overline{g}_i^{l/h}}{\partial l_j} = \begin{cases} 1/h_j, & i = j \\ 0, & i \neq j \end{cases} \qquad \frac{\partial \overline{g}_i^{l/h}}{\partial t_j} = \frac{\partial \overline{g}_i^{l/h}}{\partial d_j} = 0 \quad (67)$$

$$\frac{\partial \underline{g}_i^{d/l}}{\partial h_j} = \frac{\partial \underline{g}_i^{d/l}}{\partial t_j} = 0 \qquad \frac{\partial \underline{g}_i^{d/l}}{\partial l_j} = \begin{cases} d_j/l_j^2, & i = j \\ 0, & i \neq j \end{cases} \qquad \frac{\partial \underline{g}_i^{d/l}}{\partial d_j} = \begin{cases} -1/l_j, & i = j \\ 0, & i \neq j \end{cases} \quad (68)$$

$$\frac{\partial \overline{g}_i^{d/l}}{\partial h_j} = \frac{\partial \overline{g}_i^{d/l}}{\partial t_j} = 0 \qquad \frac{\partial \overline{g}_i^{d/l}}{\partial l_j} = \begin{cases} -d_j/l_j^2, & i = j \\ 0, & i \neq j \end{cases} \qquad \frac{\partial \overline{g}_i^{d/l}}{\partial d_j} = \begin{cases} 1/l_j, & i = j \\ 0, & i \neq j \end{cases} \quad (69)$$

After calculating the gradients above, Eqns. (62), and (65)-(69) are used to guide the search direction of the gradient-based optimization scheme described in the following section.

S7-G. Optimization method

Eqn. (61) describes a nonlinear optimization problem with multiple equality and inequality constraints. To solve it, we opt for the Powell-Hestenes-Rockafellar (PHR) method[①], an Augmented Lagrangian algorithm well suited for minimization problems with equality and inequality constraints. The Lagrange function defined for the primal problem is reframed with the definition of a set of penalty functions, which

[①] Rockafellar, R. T. The multiplier method of Hestenes and Powell applied to convex programming. *Journal of Optimization Theory and applications* **12**, 555-562 (1973).

allow transforming the primal problem into a series of unconstrained sub-problems. The Augmented Lagrangian function corresponding to the primal problem in Eqns. (61) is constructed as:

$$\psi(\boldsymbol{X},\boldsymbol{\mu},\boldsymbol{\lambda},\boldsymbol{\sigma}) = F - \sum_{i=1}^{m} \mu_i g_i + \frac{\sigma}{2}\sum_{i=1}^{m} g_i^2 + \frac{1}{2\sigma}\sum_{\iota=1}^{4m}([\min\{0, -\sigma g_\iota^I - \lambda_\iota\}]^2 - \lambda_\iota^2) \tag{70}$$

where $\boldsymbol{X}$ is a vector representing the design variables (Eqns. (53)), $\boldsymbol{\mu}$ and $\boldsymbol{\lambda}$ are the Lagrange multipliers, and $\boldsymbol{\sigma}$ the penalty multiplier; g_ι^I represents the ι- th inequality constraint, including $\underline{g}_i^{l/h}, \overline{g}_i^{l/h}, \underline{g}_i^{d/l}$, and $\overline{g}_i^{d/l}$. In the k- th step of the iterative process for solving the primal problem, starting from the design point, $\boldsymbol{X}^k$, the design point for the $(k+1)$-th step is obtained by minimizing the following unconstrained sub-problem:

$$\min\ \psi(\boldsymbol{X}, \boldsymbol{\mu}^k, \boldsymbol{\lambda}^k, \sigma^k) \tag{71}$$

To solve Eqn. (71), we resort to the Broyden-Fletcher-Goldfarb-Shanno (BFGS) method① that updates the design variables such that the updating scheme, in the k_j-th step of the solution process of the sub-problem, is given by:

$$X_{m_j}^{k_j+1} = \begin{cases} X_{m_j}^L & \text{if } X_{m_j}^k - \beta^{k_j} d_{m_j}^k \leq X_{m_j}^L \\ X_{m_j}^k - \beta^{k_j} d_{m_j}^k & \text{if } X_{m_j}^L < X_{m_j}^k - \beta^{k_j} d_{m_j}^k \leq X_{m_j}^U \\ X_{m_j}^U & \text{if } X_{m_j}^U < X_{m_j}^k - \beta^{k_j} d_{m_j}^k \end{cases} \tag{72}$$

where $X_{m_j}^{k_j}$ is the m_j-th component in the design variable vector $\boldsymbol{X}^{k_j}$ ($m_j = 1,2,\dots,4m$), β^{k_j} is the step size in the k_j-th step, $X_{m_j}^L$ and $X_{m_j}^U$ are the lower and upper bounds on the design variable $X_{m_j}^{k_j}$, respectively, and $d_{m_j}^{k_j}$ is the m_j-th component in the search direction $\boldsymbol{d}^{k_j}$, which is determined by

$$\boldsymbol{d}^{k_j} = \boldsymbol{V}^{k_j} \boldsymbol{\nabla} \psi^{k_j} \tag{73}$$

where $\boldsymbol{V}$ is the "metric-correcting transformation", which is an approximation of the inverse of the Hessian matrix, and $\boldsymbol{\nabla}\psi$ is the gradient vector of the Augmented Lagrangian function in Eqn. (70). The components of $\boldsymbol{\nabla}\psi$ are then expressed as

① Gill, P. E., Murray, W. & Wright, M. H. *Practical optimization*. (Academic Press, 1981).

$$\nabla\psi = \begin{bmatrix} \frac{\partial F}{\partial h_j} + \sum_{i=1}^{m}(\sigma g_i - \mu_i)\frac{\partial g_i}{\partial h_j} + \sum_{\iota=1}^{4m}(\min\{0, -\sigma g_\iota^E - \lambda_\iota\})\frac{\partial g_\iota^I}{\partial h_j} \\ \frac{\partial F}{\partial t_j} + \sum_{i=1}^{m}(\sigma g_i - \mu_i)\frac{\partial g_i}{\partial t_j} + \sum_{\iota=1}^{4m}(\min\{0, -\sigma g_\iota^E - \lambda_\iota\})\frac{\partial g_\iota^I}{\partial t_j} \\ \frac{\partial F}{\partial l_j} + \sum_{i=1}^{m}(\sigma g_i - \mu_i)\frac{\partial g_i}{\partial l_j} + \sum_{\iota=1}^{4m}(\min\{0, -\sigma g_\iota^E - \lambda_\iota\})\frac{\partial g_\iota^I}{\partial l_j} \\ \frac{\partial F}{\partial d_j} + \sum_{i=1}^{m}(\sigma g_i - \mu_i)\frac{\partial g_i}{\partial d_j} + \sum_{\iota=1}^{4m}(\min\{0, -\sigma g_\iota^E - \lambda_\iota\})\frac{\partial g_\iota^I}{\partial d_j} \end{bmatrix} \quad (j = 1,2,\ldots,m) \quad (74)$$

Using the BFGS method, the updating formula for the approximation of the Hessian matrix is of the form

$$\boldsymbol{V}^{k_j+1} = \left[\mathbf{I} - \frac{\Delta\boldsymbol{X}^{k_j}\left(\Delta\boldsymbol{\delta}^{k_j}\right)^{\mathrm{T}}}{\left(\Delta\boldsymbol{X}^{k_j}\right)^{\mathrm{T}}\Delta\boldsymbol{\delta}^{k_j}}\right]\boldsymbol{V}^{k_j}\left[\mathbf{I} - \frac{\Delta\boldsymbol{X}^{k_j}\left(\Delta\boldsymbol{\delta}^{k_j}\right)^{\mathrm{T}}}{\left(\Delta\boldsymbol{X}^{k_j}\right)^{\mathrm{T}}\Delta\boldsymbol{\delta}^{k_j}}\right] + \frac{\Delta\boldsymbol{X}^{k_j}\left(\Delta\boldsymbol{X}^{k_j}\right)^{\mathrm{T}}}{\left(\Delta\boldsymbol{X}^{k_j}\right)^{\mathrm{T}}\Delta\boldsymbol{\delta}^{k_j}} \quad (75)$$

where $\Delta\boldsymbol{X}^{k_j} = \boldsymbol{X}^{k_j+1} - \boldsymbol{X}^{k_j}$ and $\Delta\boldsymbol{\delta}^{k_j} = \nabla\psi^{k_j+1} - \nabla\psi^{k_j}$. To determine the appropriate step size during the search for an improved point, whereby the design objective moves towards a feasible descent direction, we define and minimize the following descent function:

$$\phi^{k_j} = \psi\left(\boldsymbol{X}^{k_j}, \boldsymbol{\mu}^k, \boldsymbol{\lambda}^k, \sigma^k\right) \quad (76)$$

A sequence of trial step size, s_J, is defined as

$$s_J = (0.5)^J \qquad J = 0,1,2,3,4,\ldots \quad (77)$$

At the $k_j - th$ iteration, we determine an acceptable size as β^{k_j}=s_J, with J as the smallest integer to satisfy the descent condition

$$\psi\left(\boldsymbol{X}^{k_j} - s_J\boldsymbol{V}^{k_j}\nabla\psi^{k_j}, \boldsymbol{\mu}^k, \boldsymbol{\lambda}^k, \sigma^k\right) \le \phi^{k_j} - s_J\gamma^{k_j} \quad (78)$$

The constant γ^{k_j} is determined using the search direction $\gamma^{k_j} = \tau\left\|\boldsymbol{V}^{k_j}\nabla\psi^{k_j}\right\|^2$, where τ is a specified constant between 0 and 1.

After solving the subproblem Eqn. (71), the design point $\boldsymbol{X}^{k+1}$ is obtained for the $(k+1)$-th step in the iterating process solving the primal problem, while the multipliers are updated through the following rule:

$$\left(\mu^{k+1}\right)_i = \left(\mu^k\right)_i - \sigma g_i\left(\boldsymbol{X}^k\right), \qquad i = 1,2,\ldots,m \quad (79)$$

$$\left(\lambda^{k+1}\right)_i = \max\left\{0, \left(\lambda^k\right)_\iota + g_\iota^I\left(\boldsymbol{X}^k\right)\right\}, \qquad \iota = 1,2,\ldots,4m \quad (80)$$

$$\sigma^{k+1} = \begin{cases} \eta\sigma^k, & \text{if } \rho^k \ge \vartheta\rho^{k-1} \\ \sigma^k, & \text{if } \rho^k < \vartheta\rho^{k-1} \end{cases} \quad (81)$$

where

$$\rho^k = \left(\sum_{i=1}^{m} g_i^2(\boldsymbol{X}^k) + \sum_{\iota}^{4m} \left[\min\left\{ -g_\iota^I(\boldsymbol{X}^k), \frac{(\lambda^k)_i}{\sigma} \right\} \right]^2 \right)^{1/2} \tag{82}$$

ϑ is a specified constant between 0 and 1, and η is larger than 1.

The design problem in Eqns. (61) is solved iteratively until the following set of convergence criteria is satisfied:

$$\begin{cases} \left|F^{k+1} - F^k\right| \leq \varepsilon_1 \text{ or } \left\|\boldsymbol{X}^{k+1} - \boldsymbol{X}^k\right\| \leq \varepsilon_2 \\ max\{\left|g_1^{k+1}\right|, \dots, \left|g_m^{k+1}\right|, \left|g_1^{I,k+1}\right|, \dots, \left|g_{4m}^{I,k+1}\right|\} \leq \varepsilon_3 \end{cases} \tag{83}$$

where ε_1 , ε_2 and ε_3 are convergence control parameters taking respectively the value of 0.001, 0.001, and 0.01.

S8. Implementation of morphing on target scheme

This section shows the implementation of the inverse problem scheme explained in S7 to the solution of the last two illustrative demonstrations of morphing on target. While they both share a primary objective, the central axis of the target domain (i.e. an "M"), their boundaries are distinct. The first assumes a uniform, yet unspecified value of the width, which is to find, and the second case features a varying width to match. In both cases, the constituent solids are silicone rubber and wood with CTE and elastic properties reported in section S2.

S8-A. Demonstration 1: uniform width

As a morphing target, we chose the profile of an "M", and use an arc spline composing smooth, G^1 continuous, arcs to prescribe its central axis (Supplementary Fig. S16). Because $x = 7.4$ is a vertical axis of symmetry, only half "M" is here examined. The width of the "M" boundary is uniform along the "M", meaning that $l_1 + 2t_0 = l_2 + 2t_0 = \cdots = l_m + 2t_0$ for all BBs. The central axis of the half domain is here mathematically expressed piece-wisely by the following set of primitives (Supplementary Fig. S16), each bounded by a pair of blending points:

$$f(x,y) = \begin{cases} f_1(x,y) = (y-1.4)^2 + x^2 - 1.4^2 = 0 & \text{if } 0 \leq x < 1.4 & 0 \leq y < 1.6 \\ f_2(x,y) = (y-2.8)^2 + (x-14.2)^2 - 12.8^2 = 0 & \text{if } 1.4 \leq x < 3.2 & 1.6 \leq y < 9.4 \\ f_3(x,y) = (y-8.9)^2 + (x-4.1)^2 - 1.0^2 = 0 & \text{if } 3.2 \leq x < 4.8 & 9.4 \leq y < 9.6 \\ f_4(x,y) = (y-7.7)^2 + (x-2.9)^2 - 2.7^2 = 0 & \text{if } 4.8 \leq x < 5.6 & 8.1 < y \leq 9.6 \\ f_5(x,y) = (y-8.6)^2 + (x-8.6)^2 - 3.1^2 = 0 & \text{if } 5.6 \leq x < 6.5 & 6.3 < y \leq 8.1 \\ f_6(x,y) = (y-7.3)^2 + (x-7.4)^2 - 1.3^2 = 0 & \text{if } 6.5 \leq x \leq 7.4 & 6.0 \leq y \leq 6.3 \end{cases} \tag{84}$$

As per Eqn. (53), we look for the optimum values of the design variables h_i, t_i, l_i, d_i $(i = 1,2, \dots, m)$. Because the width of the building block, i.e. $\omega_i = l_i + 2t_0$, is prescribed as uniform along the central axis of the target domain and t_0 is constant, $l_1 = l_2 = \cdots = l_i = \cdots = l_m$ and the objective function reduces to

the sum of squares of Eqn. (50). For a given number of building blocks (here chosen as 50), the problem formulation (Eqns. (61)) is abridged to

$$\begin{cases} \min\limits_{h_i,t_i,l_i,d_i} \quad F = \sum_{i=1}^{50} \|\boldsymbol{p}_i' - \boldsymbol{r}_i'\|^2 \\ \text{s.t.} \quad g_i = 0, \\ 4 \le l_i/h_i \le 10, \\ 0.01 \le d_i/l_i \le 0.6, \\ 0.05 \le h_i \le 5, \\ 0.05 \le t_i \le 5, \\ 0.05 \le l_i \le 5, \\ 0.005 \le d_i \le 5, \quad i = 1,2,\ldots,50 \end{cases} \tag{85}$$

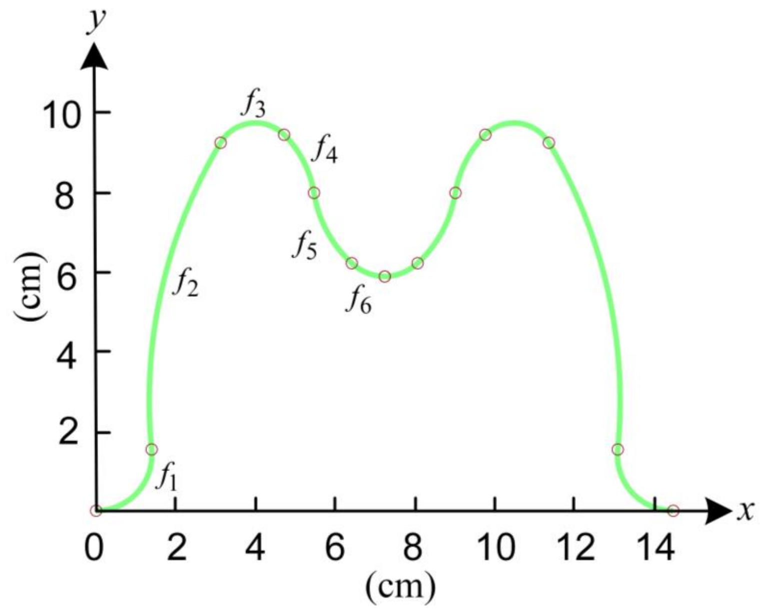

Supplementary Fig. S16: Central axis of the target domain with arc functions and blending points given by Eqn. (84).

Supplementary Fig. S17 shows the outcome of assigning the following initial values to the design variables: $h_i = 0.1, t_i = 0.1, l_i = 0.7, d_i = 0.35, i = 1,2,\ldots,50$. The metamaterial phenotype at $T = 120$ ℃ is shown in the lower part with grey and blue representing the low CTE frame and the high CTE core of the BB sequence. On the upper part appears the target curve (green solid line) and the reference points R_i (red).

Due to the randomly assigned values of the design variables, the central axis of the metamaterial phenotype is off from the target. After application of the tailoring scheme, though, the phenotype is realigned to the target after 80 iterations. Supplementary Fig. S18 shows the iteration history for the

process to gradually converge to an optimal solution. At convergence, the objective value decreases from the initial 776 to the final 2.81×10^{-6}, a tiny value indicating the attainment of the gap closure, where the phenotype axis conforms seamlessly to the central axis of the target.

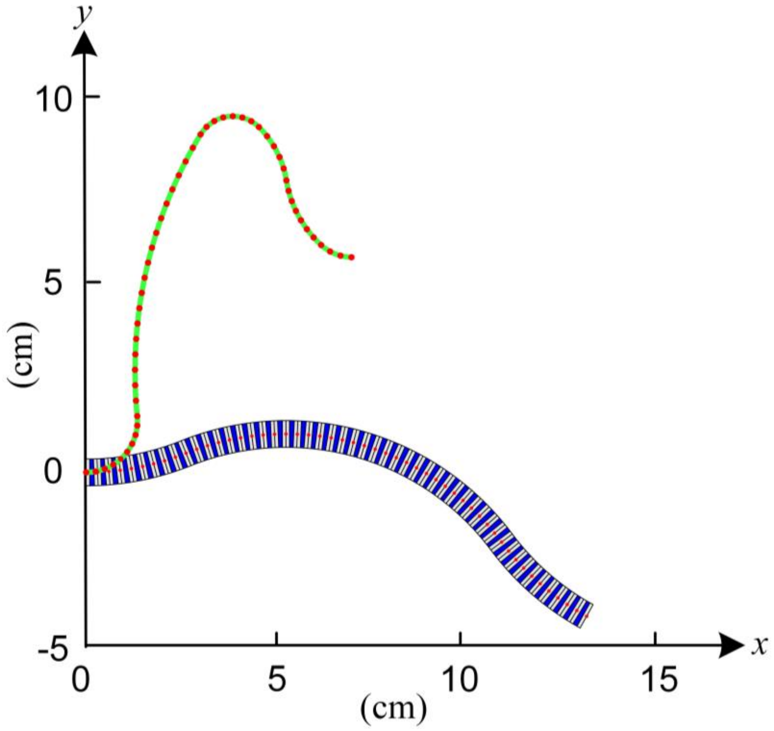

Supplementary Fig. S17: Metamaterial phenotype ($T = 120$ ℃) with initial guesses for the design variables and reference points on the target curve.

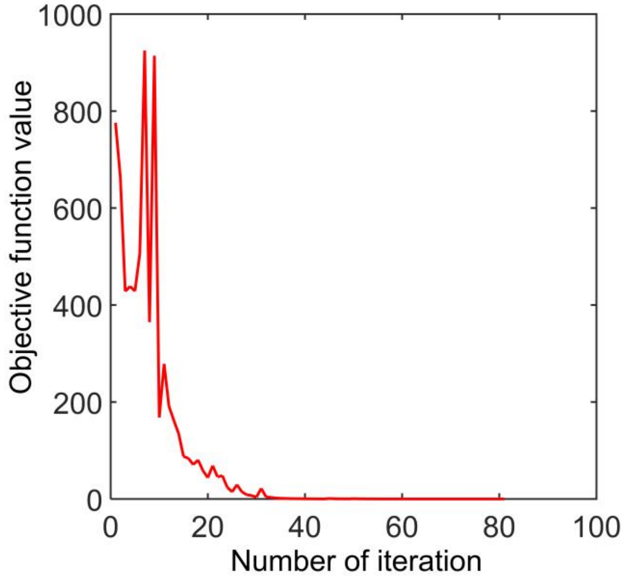

Supplementary Fig. S18: Convergence plot for the objective function.

Supplementary Fig. S19 shows, in a stem plot, the optimized values of the design variables along with their relevant ratios, which define the metamaterial genotype at the initial temperature ($T = 20$ ℃). The values shown in sequence for the 50 BBs are descriptive of each BB geometry as well as their sequence. As described in the caption of Fig.1, each building block is described with $B_{\frac{t}{h}}^{+/-\left(h,\frac{l}{h},\frac{d}{l}\right)}$, where $B = U$ for U-BB and $B = R$ for R-BB, the sign $+/-$ specifies the direction of rotation for R-BB (clockwise: $+$; counter-clockwise: $-$) and is omitted for U-BB. With this notation, we can describe the string of information for a sequence of m BBs as $B_{\frac{t_1}{h_1}}^{+/-\left(h_1,\frac{l_1}{h_1},\frac{d_1}{l_1}\right)} B_{\frac{t_2}{h_2}}^{+/-\left(h_2,\frac{l_2}{h_2},\frac{d_2}{l_2}\right)} \dots B_{\frac{t_m}{h_m}}^{+/-\left(h_m,\frac{l_m}{h_m},\frac{d_m}{l_m}\right)}$, which can be further condense to $\left(B_{\frac{t}{h}}^{+/-\left(h,\frac{l}{h},\frac{d}{l}\right)}\right)_{\eta}$ for η consecutive units with identical BB parameters. This sequence is shown on the bottom of Supplementary Fig. S19 for the "M" problem and constitutes the tailored code that enables the phenotype to morph on target.

Supplementary Fig. S20 shows the resulting configuration at $T = 120$ ℃ along with the phenotype axis and the target axis. The similarity between the phenotype axis and the target axis is evaluated by calculating the coefficient of determination R^2, which is mathematically defined as

$$R^2 = 1 - \frac{\sum_{i=1}^{m}\left[\left(x_{pi} - x_{ri}\right)^2 + \left(y_{pi} - y_{ri}\right)^2\right]}{\sum_{i=1}^{m}\left[\left(x_{pi} - \bar{x}_p\right)^2 + \left(y_{pi} - \bar{y}_p\right)^2\right]} \tag{86}$$

where (x_{pi}, y_{pi}) and (x_{ri}, y_{ri}) represent the coordinates of the BB interface point i of the phenotype on target and the corresponding reference point on the target axis respectively. $\bar{x}_p$ and $\bar{y}_p$ denote the mean value of the relevant coordinates for BB interface points. The coefficient of determination indicates the precision of the fit for both x- and y-values and ranges from 0 to 1. The better the match between phenotype and target, the closer the value of R^2 to 1. In our example, the value of R^2 is 0.994, meaning a remarkably good agreement between target and predicted phenotype axis. A minor discrepancy appears (Supplementary Fig. S20) with the reason attributed to the adoption of a surface response for approximating the opening angle of each R-BB (see S3-C-i).

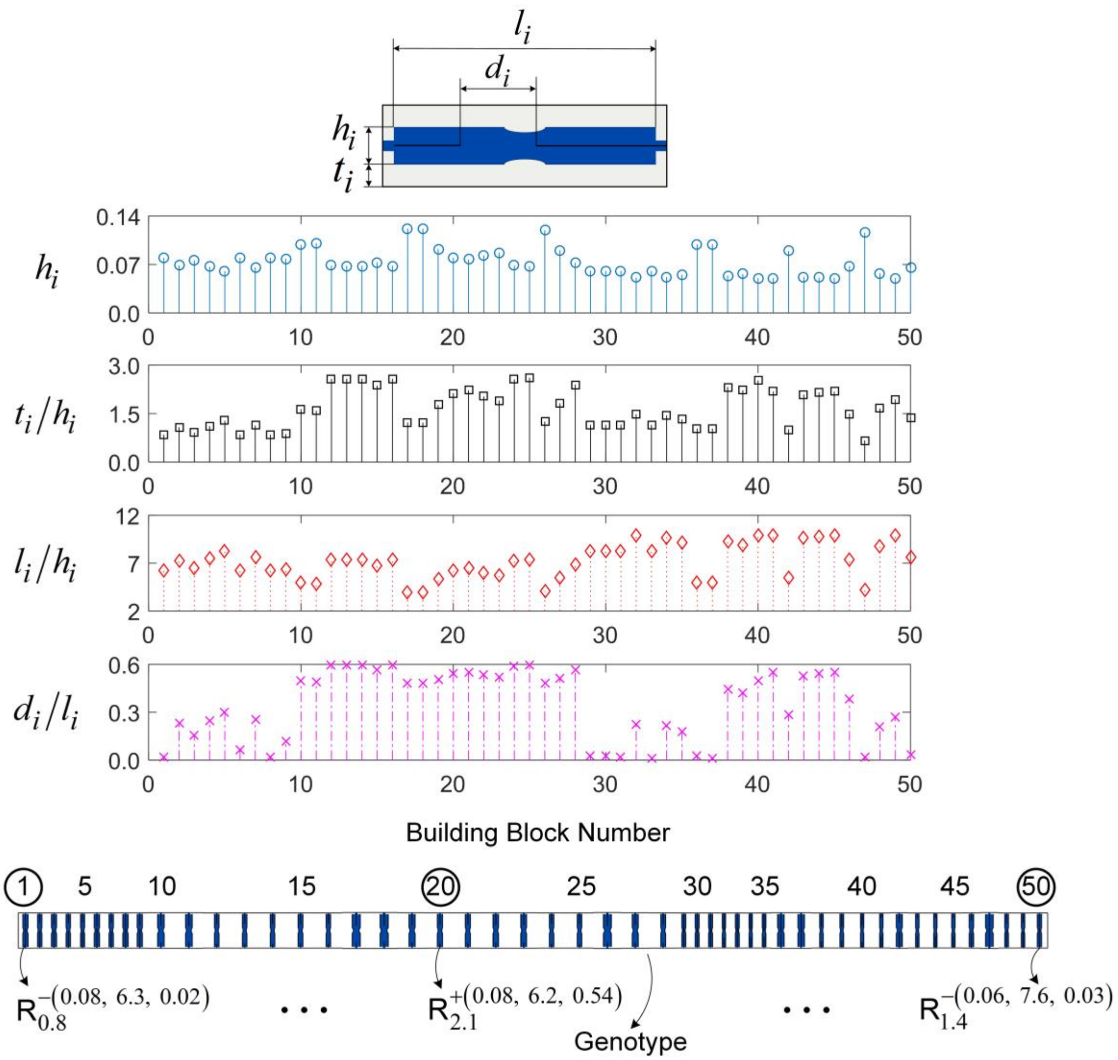

Supplementary Fig. S19: BB geometry with 4 sets of values for the design variables optimized to match the target central axis. The values provide the full geometric description to each of the 50 units making up the metamaterial genotype, which is shown below in its initial undeformed state. Below the genotype is the reported BB sequence code given only for BB_1, BB_{20} and BB_{50}, chosen as representative BBs.

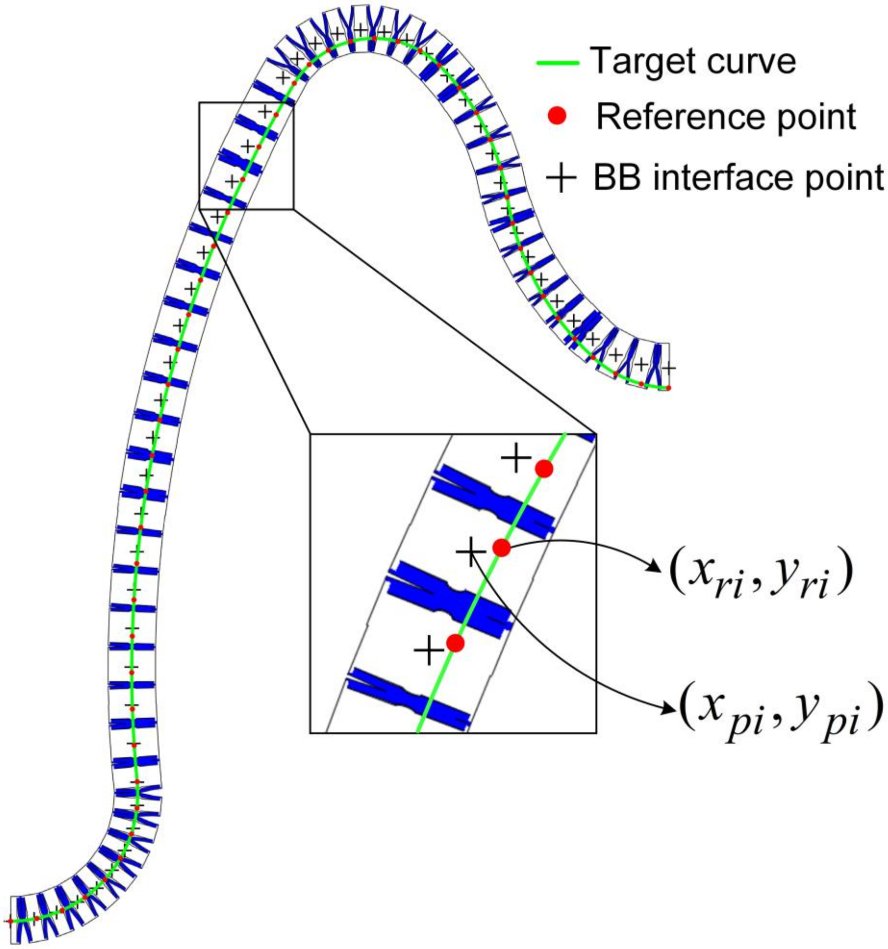

Supplementary Fig. S20: Predicted phenotype at $T = 120$ ℃ along with BB interface point (black cross), the target axis (green), and reference point (red). The assessment of the match is given by the coefficient of determination, R^2= 0.994 in this case, with unity indicating a perfect match between the two.

S8-B. Demonstration 2: varying width

In this example, the central axis of the target domain has the "M" shape of that in Case study 1, hence expressed by Eqn. (84). But the width of the domain boundary is not uniform and specified to vary along the target axis (Fig.5b and Supplementary Fig. S21). Due to symmetry about $x = 7.4$, half of the domain is examined with the target width piece-wisely expressed in the arc-length coordinate system by:

$$w(s) = \begin{cases} w_1(s) = -2.86 \times s + 0.90 & 0.00 \le s < 0.14 \\ w_2(s) = 1.13 \times s + 0.34 & 0.14 \le s < 0.67 \\ w_3(s) = -2.78 \times s + 2.96 & 0.67 \le s < 0.85 \\ w_4(s) = 1.33 \times s - 0.53 & 0.85 \le s \le 1.00 \end{cases} \quad (87)$$

where s denotes the normalized arc length and is defined as:

$$s(x,y) = \int_0^x \sqrt{1 + \left(\frac{\partial f/\partial x}{\partial f/\partial y}\right)^2}\, dx \Bigg/ \int_0^{x_{end}} \sqrt{1 + \left(\frac{\partial f/\partial x}{\partial f/\partial y}\right)^2}\, dx \tag{88}$$

where $f(x,y)$ is the mathematical expression of the central axis of the target, i.e. Eqn. (84), and x_{end} represents the x −coordinate of the end point of half domain. Supplementary Fig. S21 shows both the central axis (green) and varying width (shaded areas of dissimilar colors) of the target domain.

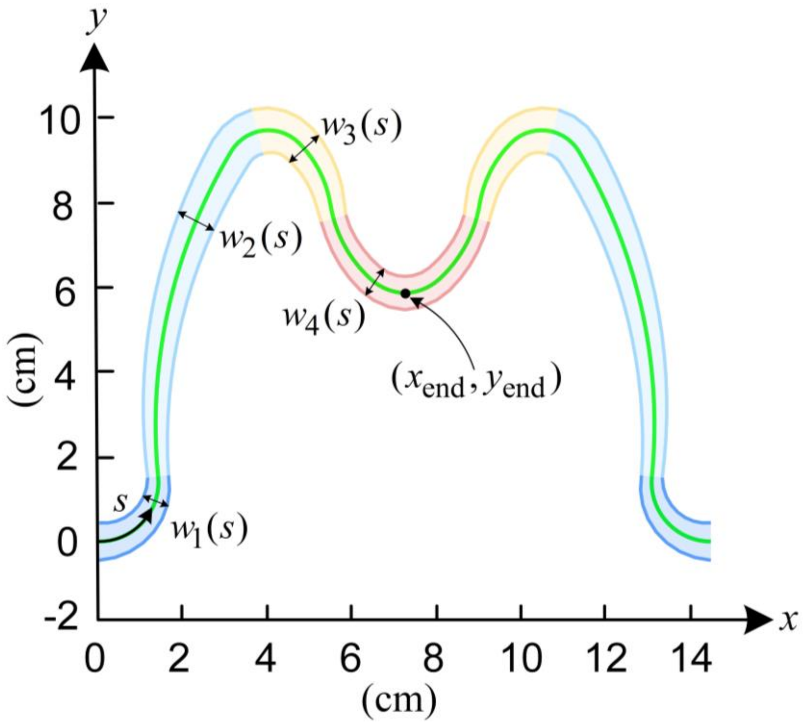

Supplementary Fig. S21: Central axis of the target domain along with its varying widths (4 arc primitives for half domain) defined by Eqn. (87).

As per Eqn. (53), we look for the optimum values of the design variables h_i, t_i, l_i, d_i $(i = 1,2, \dots, m)$. Since both the axis and width functions of the target are specified, the problem is formulated for a given number of building blocks (here chosen to be 46) as:

$$\begin{cases} \min\limits_{h_i,t_i,l_i,d_i} \quad F = \sum_{i=1}^{46} [\|\boldsymbol{p_i} - \boldsymbol{r_i}\|^2 + |\omega_i - w(\boldsymbol{r_i})|^2] \\ \text{s.t.} \quad g_i = 0, \\ 4 \le l_i/h_i \le 10, \\ 0.01 \le d_i/l_i \le 0.6, \\ 0.05 \le h_i \le 5, \\ 0.01 \le t_i \le 5, \\ 0.05 \le l_i \le 5, \\ 0.005 \le d_i \le 5, \quad i = 1,2, \dots, 46 \end{cases} \tag{89}$$

The optimization is implemented under the following initial values of the design variables: $h_i = 0.1, t_i = 0.1, l_i = 0.7, d_i = 0.35, i = 1,2, \ldots, 46$. The tailoring process gradually converges to an optimal solution after 128 iterations, and Supplementary Fig. S22 shows the iteration history for the objective function. At convergence, the objective value decreases from the initial 2024 to the final 1.82×10^{-5}, an extremely small value indicating the achievement of the gap closure, where the phenotype axis and its width conform seamlessly to the axis and boundary of the target.

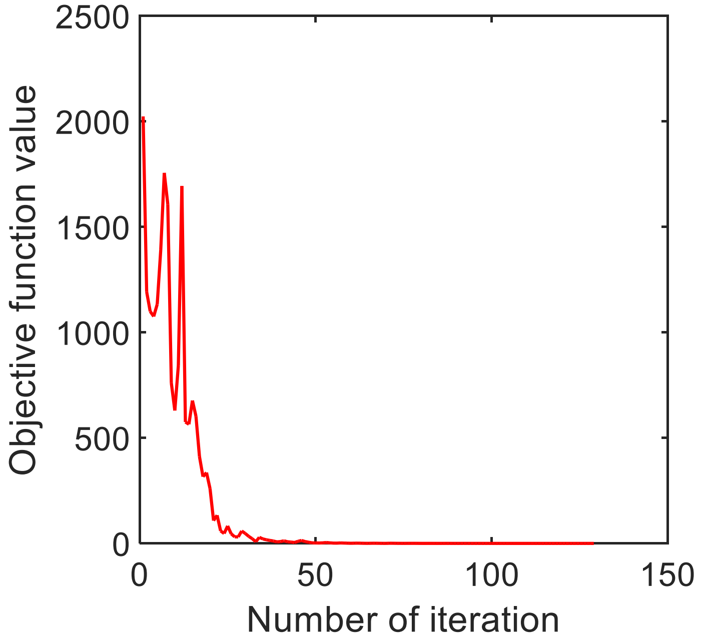

Supplementary Fig. S22: Convergence plot for the objective function.

Fig.5b shows in a stem plot the optimized values of the design variables along with the relevant ratios, which define the metamaterial genotype at the initial temperature ($T = 20$ ℃). The values shown in sequence for the 46 BBs are descriptive of each BB geometry. Their use in the construction of the genotype is shown below along with a representative set of the BB sequence code, used in turn to computationally predict the on-target phenotype shown in Supplementary Fig. S23. Here shown is the resulting configuration at $T = 120$ ℃ along with the phenotype axis and the target axis. The coefficient of determination R^2, as defined in Eqn. (86), is used to assess the deviation of the phenotype axis from the target axis with a value of 0.997, indicating a remarkably good match. The reason for the difference between central axis target and predictions can be attributed to the use of a surface response for the opening angle. As for the matching of the varying width of the target domain, i.e. ($\omega_i = l_i + 2t_0$), which is explicit to the design variables, there exists no deviation.

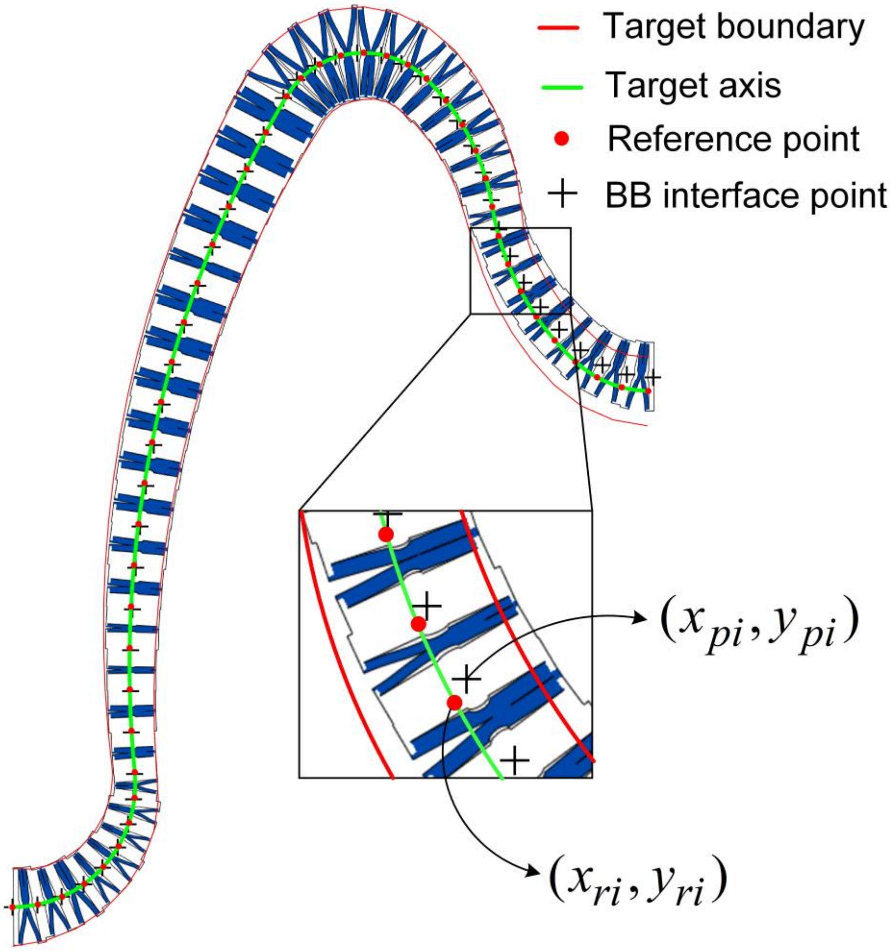

Supplementary Fig. S23: Predicted phenotype at $T = 120$ ℃ along with BB interface point (black cross), the target axis (green) and boundary (red). The precision of the match is R^2=0.997, a value approaching unit, the perfect match.
